# Global, regional, and national burden and trends analysis of gallbladder and biliary tract cancer from 1990 to 2019 and predictions to 2030: a systematic analysis for the Global Burden of Disease Study 2019

**DOI:** 10.3389/fmed.2024.1384314

**Published:** 2024-04-04

**Authors:** Jiao Su, Yuanhao Liang, Xiaofeng He

**Affiliations:** ^1^Department of Biochemistry, Changzhi Medical College, Changzhi, China; ^2^Clinical Experimental Center, Jiangmen Key Laboratory of Clinical Biobanks and Translational Research, Jiangmen Central Hospital, Jiangmen, China; ^3^Institute of Evidence-Based Medicine, Heping Hospital Affiliated to Changzhi Medical College, Changzhi, China

**Keywords:** gallbladder and biliary tract cancer, disease burden, epidemiology, ASR, EAPC

## Abstract

**Objectives:**

Our aim was to explore the disease burden caused by gallbladder and biliary tract cancer globally, regionally, and nationally, by age and sex.

**Methods:**

The absolute number of cases and age-standardized rates (ASR) of incidence, prevalence, mortality, and disability-adjusted life years (DALYs) due to gallbladder and biliary tract cancer were extracted from the Global Burden of Disease (GBD) Study 2019. We estimated the trends in disease burden by calculating the percentage change in the absolute number of cases and the estimated annual percentage change (EAPC) in ASR, by social development index (SDI), region, nation, sex, and age.

**Results:**

From 1990 to 2019, the number of incident cases, prevalent cases, deaths, and DALYs worldwide significantly increased by 1.85-fold, 1.92-fold, 1.82-fold, and 1.68-fold, respectively. However, the age-standardized rates of incidence, prevalence, mortality, and DALYs tend to decrease globally over time. Nevertheless, heterogeneous disease burden patterns exist between geographic regions due to different geographical risk factors, distinct epidemiologically predominant gallbladder and biliary tract cancer subtypes, and potential genetic predispositions or ethnicity. Additionally, socioeconomic status mediates the regional variation in disease burden, with increasing SDI or HDI scores associated with downward trends in the age-standardized rates of incidence, prevalence, mortality, and DALYs. Older individuals and females are at higher risk of gallbladder and biliary tract cancer, but the increasing burden of early-onset gallbladder and biliary tract cancer is a cause for concern, especially for those living in lower SDI areas and males. High BMI is the primary risk factors underlying gallbladder and biliary tract cancer, accounted for 15.2% of deaths and 15.7% DALYs globally in 2019.

**Conclusion:**

Our study comprehensively elucidated the distribution and dynamic trends of gallbladder and biliary tract cancer burden over the past three decades, from multiple dimensions. These findings emphasize the importance of promoting a healthy lifestyle as a population-level cancer prevention strategy and tailoring cancer control actions based on localized risk factors and the epidemic profiles of gallbladder and biliary tract cancer by anatomical subtype.

## 1 Introduction

Gallbladder and biliary tract cancers are insidiously aggressive and malignant tumors, carrying a grave prognosis (1). Due to the absence of typical clinical signs or symptoms in most patients at an early stage, diagnosis often occurs at an advanced stage, rendering less than 20% of patients eligible for complete surgical resection, the currently most effective curative treatment available (2). The mean survival time for patients in the late stages is approximately 6 months, with an overall 5-year survival rate of less than 5% (3). In 2017, an estimated 210,878 new cases and 173,974 deaths were reported worldwide, ranking these cancers as the 22nd and 16th most common among 33 cancers, respectively (4, 5). From 1990 to 2017, globally there was a significant increase of 76.0% in the number of incident cases and a 65% increase in the number of deaths related to gallbladder and biliary tract cancers. However, it is worth noting that the overall age-standardized rate of incidence and mortality of these cancers have actually decreased, with a decline of 14 and 20%, respectively (5). In general, there is substantial variation in the incidence and mortality rates of gallbladder and biliary tract cancers across countries and regions, with higher rates generally observed in Asia and South America compared to Europe and North America (6, 7). Additionally, despite the mortality rates from gallbladder and biliary tract cancers are on the decline in the majority of countries worldwide, some high-income countries are experiencing an increase in mortality rates after decades of decline (6). This changing survival pattern can be attributed to several factors, such as unhealthy lifestyle, chronic diseases, and the aging of populations (8), highlighting the need for close monitoring of the burden of disease and the impact of modifiable risk factors.

Gallbladder and biliary tract cancers pose a significant public health concern, displaying distinct variations in epidemiology and risk factors across different geographical regions and ethnicity (9). These geodemographic disparities in the incidence and prevalence of gallbladder and biliary tract cancers can be attributed to various factors, such as differences in dietary structure, nutritional status, the prevalence of gallstones and obesity, socioeconomic status, as well as environmental and genetic factors (10, 11). Moreover, both women and the elderly exhibit a higher susceptibility to gallbladder and biliary tract cancers (11, 12). Given the global unmet need for prevention and treatment strategies, a comprehensive and up-to-date assessment of the disease burden caused by gallbladder and biliary tract cancers, considering regional, temporal, gender, and age variations, is essential for informing policy-making and resource allocation.

The GBD Study systematically quantifies the incidence, prevalence, mortality, and resulting health loss caused by diseases and injuries (13). In this study, we utilized data from the GBD study 2019 to provide global, regional, and national-level estimates of the incidence, prevalence, mortality, and disability-adjusted life years (DALYs) associated with gallbladder and biliary tract cancers from 1990 to 2019, considering variations in region, time, gender, age, and the Socio-Demographic Index (SDI). These updated estimates shed light on the spatial, temporal, and geodemographic distribution of gallbladder and biliary tract cancers and their associated disease burden, thus enhancing our understanding of the evolving challenges and providing scientific evidence to optimize preventive strategies and prioritize health resource allocation.

## 2 Methods

### 2.1 Data source and data collection

The GBD study 2019, led by the Institute for Health Metrics and Evaluation (IHME), aims to assess the comparative magnitude of health loss caused by diseases, injuries, and risk factors across different age groups, sexes, and geographic regions at specific points in time in 204 countries and territories. The GBD study has been updated annually since 1990, employing a standardized approach to generate regular estimates of disease burden in terms of incidence, prevalence, mortality, years of life lost (YLLs), years lived with disability (YLDs), and DALYs for various causes, risk factors, and combinations thereof. DALYs represent the sum of years of potential life lost due to premature death (YLLs) and years of productive life lost due to disability (YLDs), compared to a standardized life expectancy (14). This measure provides a comprehensive assessment of disease burden, encompassing the cumulative number of years lost to ill-health, disability, or premature death. Detailed information about the GBD protocol can be accessed online.^1^ We collected annual incidence, age-standardized incidence rate, prevalence, age-standardized prevalence rate, mortality, age-standardized mortality rate, DALYs, age-standardized DALYs rate, YLLs, age-standardized YLLs rate, YLDs, and age-standardized YLDs rate for gallbladder and biliary tract cancers from 1990 to 2019, categorized by sex, region, and country, from the Global Health Data Exchange (GHDx) query tool.^2^ Moreover, we obtained data on the burden of early-onset gallbladder and biliary tract cancers, defined as cancers diagnosed in adults under the age of 50 (15), from the GBD study 2019.

In the GBD study, gallbladder and biliary tract cancers are identified by the International Classification of Diseases 10th revision (ICD-10) C23, C24-C24.9. In 2019, there were globally 256,340 (95% uncertainty interval [UI]: 215,699 to 282,004) people suffering from gallbladder and biliary tract cancers, with 199,211 (95% UI, 166,769-219,615) newly diagnosed cases in the same year. Furthermore, the Socio-demographic Index (SDI) developed by GBD researchers consists of three metrics: the total fertility rate under the age of 25, the mean education for those ages 15 and older, and the lag distributed income *per capita*. This index serves as a composite indicator of development status that is strongly correlated with health outcomes. Based on the score of the socio-demographic index (SDI), which is calculated as geometric mean ranging from 0 to 1, regions and countries are categorized into five quintiles: low, low-middle, middle, high-middle, and high. Additionally, the human development index (HDI), which can be accessed at the United Nations Development Program^3^ is commonly used to assess the level of human development in countries across three fundamental dimensions: life expectancy, accessibility of education and literacy, and living conditions and income. The HDI is divided into four levels: very high human development (0.8–1.0), high human development (0.7–0.79), medium human development (0.55–0.70), and low human development (below 0.55) (16).

### 2.2 Statistical analysis

In this study, the age-standardized rate (ASR) and estimated annual percentage change (EAPC) were employed to measure the trends in disease burden due to gallbladder and biliary tract cancers (17). These measures provide a standardized assessment of the disease burden across different populations. Standardization is necessary when comparing multiple populations with varying age structures or when examining changes within a single population over time, as age profiles fluctuate accordingly. The ASR (per 10^5^ population) was calculated using the following formula, where $a_{i}$ denotes the age-specific rate in the $i^{th}$ age subgroup and $w_{i}$ represents the number of individuals in the same age class of the selected reference standard population.


$$\mathrm{ASR}=\frac{\sum_{i=1}^{A}a_{i}w_{i}}{\sum_{i=1}^{A}w_{i}}\times 100,000$$

Moreover, EAPC was widely applied to quantify and summarize the temporal trend of ASR (18). A regression line was fitted to the natural logarithm of the rates, denoted as y=α+βx+ε , where y=ln(ASR) , and x=calendaryear . The EAPC was calculated as 100×(exp(β)−1) , and the corresponding 95% confidence interval (CI) could be obtained from the linear regression model. If both the EAPC value and the lower limits of its 95% CI were greater than 0, the ASR was considered to exhibit an increasing trend; conversely, if both were less than 0, a decreasing trend was observed. Additionally, Pearson correlation analysis was conducted to assess the association between EAPCs and ASRs (1990), as well as HDI (2019), in order to explore the influential factors for EAPCs. In the current study, countries with missing data were excluded for the correlation analysis. What’s more, a hierarchical cluster analysis was performed to categorize countries and territories into four groups (Significant decrease; Moderate decrease; Minor decrease; and Remained stable or increase) based on the temporal trend of ASR at the national level (19).

Furthermore, the incidence and mortality of the disease burden for gallbladder and biliary tract cancers from 2020 to 2030 were projected by used the Bayesian age-period-cohort (BAPC) model integrating nested Laplace approximations (20). The BAPC model is commonly used to analyze and project age-stratified cancer incidence and mortality rates, especially in light of significant demographic changes. The R package “BAPC” streamlines the implementation of the BAPC model, allowing for the generation of well-calibrated probabilistic forecasts with relatively narrow uncertainty ranges. Standard Populations data came from the World (WHO 2000–2025) Standards database,^4^ and population forecast data was collected from the World Population Prospects 2022 that published by the United Nations Department of Economic and Social Affairs.^5^

All statistics Data analysis was conducted using R software, version 4.1.0 (R Foundation for Statistical Computing). A two-tail *p* value <0.05 was considered statistically significant.

## 3 Results

### 3.1 Global and regional levels of gallbladder and biliary tract cancers burden

Globally, the number of incident cases increased by 84.8% from 107,787 (95% UI: 96,900-119,860) in 1990 to 199,211 (95% UI: 166,769-219,615) in 2019. However, the overall age-standardized incidence rate showed a decrease of 0.48% (95% confidence interval [CI]: 0.40 to 0.55%) per year from 1990 to 2019 (Table 1; Supplementary Table S1). In 2019, there were 256,340 (95% UI: 215,699 to 282,004) prevalent cases globally, with an age-standardized prevalence rate of 3.2 (95% UI: 2.7 to 3.5) per 10^5^ population (Supplementary Table S2). The age-standardized prevalence rate decreased by an average of 0.27% (95% CI: 0.19 to 0.35%) per year from 1990 to 2019 worldwide (Table 1). Gallbladder and biliary tract cancers caused 172,441 deaths (95% UI: 144,899 to 188,615) and 3,621,473 (95% UI: 3,102,423 to 3,969,071) DALYs worldwide in 2019 (Supplementary Tables S3, S4). In 2019, the global age-standardized mortality and DALYs rates were 2.2 (95% UI: 1.8 to 2.4) and 44.0 (95% UI: 37.6 to 48.2) per 10^5^ population, decreased by an average of 0.58 and 0.67% per year globally between 1990 and 2019, respectively (Supplementary Tables S3, S4). Additionally, in 2019, the number of DALYs can be divided into 48,437 (95% UI: 33,771 to 64,794) YLDs and 3,573,035 (95% UI: 3,063,590 to 3,916,195) YLLs, with a more rapid decline in the age-standardized rate of YLLs compared to YLDs (Supplementary Tables S5, S6).

**Table 1:** The change of GBTC cases from 1990 to 2019 in global, sexes, SDI areas, and geographic regions.

Characteristics	Incidence		Prevalence		Deaths		DALYs	
	Percentage change in absolute number (%)	EAPC No. (95% CI)	Percentage change in absolute number (%)	EAPC No. (95% CI)	Percentage change in absolute number (%)	EAPC No. (95% CI)	Percentage change in absolute number (%)	EAPC No. (95% CI)
Overall	84.79	−0.48 (−0.55–−0.4)	91.70	−0.27 (−0.35–−0.19)	81.78	−0.58 (−0.65–−0.51)	68.16	−0.67 (−0.74–−0.61)
*Sex*								
Male	113.86	0.04 (−0.06–0.15)	123.14	0.28 (0.16–0.40)	109.59	−0.07 (−0.17–0.04)	89.31	−0.20 (−0.31–−0.10)
Female	67.36	−0.82 (−0.87–−0.76)	71.57	−0.66 (−0.72–−0.60)	65.69	−0.86 (−0.97–−0.75)	54.79	−0.94 (−1.05–−0.83)
*Socio-demographic index*								
High	42.09	−1.09 (−1.14–−1.03)	52.58	−0.67 (−0.74–−0.6)	36.02	−1.38 (−1.44–−1.33)	10.79	−1.85 (−1.91–−1.8)
High-middle	69.90	−0.48 (−0.6–−0.36)	82.25	−0.13 (−0.25–0)	61.56	−0.73 (−0.86–−0.6)	48.58	−0.79 (−0.92–−0.66)
Middle	150.27	0.23 (0.11–0.34)	165.69	0.53 (0.41–0.64)	139.39	0 (−0.11–0.12)	118.15	−0.1 (−0.21–0.01)
Low-middle	171.56	0.58 (0.51–0.66)	177.97	0.74 (0.67–0.8)	169.30	0.49 (0.4–0.58)	154.41	0.52 (0.43–0.6)
Low	145.95	0.54 (0.45–0.64)	153.85	0.67 (0.58–0.76)	145.88	0.49 (0.39–0.59)	136.75	0.41 (0.3–0.52)
*Region*								
High-income Asia Pacific	81.22	−1.45 (−1.49–−1.41)	88.74	−0.97 (−1.03–−0.9)	75.60	−1.79 (−1.84–−1.74)	25.03	−2.24 (−2.3–−2.17)
Central Asia	40.00	−0.09 (−0.2–0.02)	33.33	−0.14 (−0.24–−0.03)	47.92	−0.03 (−0.15–0.09)	52.69	−0.23 (−0.35–−0.1)
East Asia	204.51	1.44 (1.02–1.86)	236.49	1.96 (1.53–2.39)	181.48	1 (0.59–1.41)	143.62	0.82 (0.42–1.22)
South Asia	233.64	0.96 (0.81–1.11)	237.61	1.15 (1.01–1.28)	229.89	0.84 (0.68–1.01)	209.09	0.92 (0.77–1.07)
Southeast Asia	153.33	0.18 (0.08–0.28)	174.51	0.56 (0.46–0.65)	136.18	−0.09 (−0.19–0.02)	116.22	−0.31 (−0.4–−0.21)
Australasia	50.00	−1.19 (−1.26–−1.12)	75.00	−0.73 (−0.82–−0.65)	47.73	−1.5 (−1.57–−1.44)	28.37	−1.68 (−1.74–−1.62)
Caribbean	12.50	−2.41 (−2.92–−1.9)	11.11	−2.31 (−2.82–−1.79)	5.19	−2.46 (−2.96–−1.95)	4.40	−2.35 (−2.86–−1.84)
Central Europe	−4.48	−1.71 (−1.76–−1.66)	−1.35	−1.44 (−1.48–−1.39)	−5.86	−1.83 (−1.89–−1.78)	−15.35	−1.88 (−1.92–−1.84)
Eastern Europe	8.51	−0.77 (−0.96–−0.57)	17.86	−0.31 (−0.45–−0.17)	2.17	−1.12 (−1.34–−0.89)	−3.43	−1.17 (−1.43–−0.91)
Western Europe	4.91	−1.63 (−1.71–−1.56)	17.42	−1.07 (−1.13–−1.01)	−3.47	−2.04 (−2.13–−1.95)	−15.47	−2.2 (−2.28–−2.12)
Andean Latin America	127.27	−0.68 (−0.8–−0.56)	150.00	−0.36 (−0.47–−0.24)	118.87	−0.92 (−1.05–−0.8)	96.27	−1.14 (−1.27–−1.01)
Central Latin America	68.57	−2.27 (−2.42–−2.12)	72.50	−2.08 (−2.23–−1.92)	63.20	−2.39 (−2.53–−2.24)	50.37	−2.45 (−2.61–−2.29)
Southern Latin America	20.45	−1.62 (−1.69–−1.55)	28.00	−1.31 (−1.38–−1.24)	16.91	−1.84 (−1.91–−1.77)	8.80	−1.86 (−1.93–−1.79)
Tropical Latin America	83.87	−1.41 (−1.48–−1.33)	88.57	−1.21 (−1.28–−1.13)	82.43	−1.52 (−1.6–−1.45)	67.48	−1.53 (−1.6–−1.46)
North Africa and Middle East	122.58	−0.39 (−0.43–−0.36)	134.29	−0.17 (−0.21–−0.13)	111.82	−0.56 (−0.6–−0.53)	100.80	−0.78 (−0.81–−0.75)
High-income North America	35.44	−0.97 (−1.02–−0.93)	50.38	−0.55 (−0.61–−0.48)	22.31	−1.37 (−1.43–−1.31)	18.97	−1.4 (−1.46–−1.33)
Oceania	133.33	−0.28 (−0.31–−0.25)	166.67	−0.17 (−0.2–−0.14)	100.00	−0.36 (−0.4–−0.33)	116.46	−0.33 (−0.36–−0.3)
Central Sub-Saharan Africa	150.00	−0.48 (−0.53–−0.42)	66.67	−0.40 (−0.47–−0.33)	96.00	−0.5 (−0.54–−0.45)	98.18	−0.55 (−0.59–−0.51)
Eastern Sub-Saharan Africa	80.00	−0.33 (−0.38–−0.29)	100.00	−0.23 (−0.29–−0.16)	93.68	−0.23 (−0.27–−0.19)	83.52	−0.52 (−0.56–−0.48)
Southern Sub-Saharan Africa	150.00	−0.04 (−0.26–0.18)	66.67	−0.03 (−0.2–0.13)	100.00	−0.01 (−0.24–0.22)	91.44	−0.02 (−0.24–0.19)
Western Sub-Saharan Africa	77.78	−0.34 (−0.42–−0.26)	88.89	−0.27 (−0.37–−0.17)	82.22	−0.36 (−0.43–−0.28)	84.24	−0.42 (−0.5–−0.35)

Across GBD regions, the highest age-standardized rates of incidence, prevalence, and mortality for Gallbladder and biliary tract cancers were reported in High-income Asia Pacific, while Southern Latin America had the highest age-standardized DALYs rate (Supplementary Tables S1–S4). From 1990 to 2019, East and South Asia had the fastest increase in age-standardized rates of Gallbladder and biliary tract cancers burden. In contrast, the Caribbean showed the largest decrease of age-standardized rates of incidence (EAPC = −2.41, 95% CI: −2.92 to −1.9), prevalence (EAPC = −2.31, 95% CI: −2.82 to −1.79), and mortality (EAPC = −2.46, 95% CI: −2.96 to −1.95). However, the largest decrease in age-standardized DALYs rate was observed in Central Latin America (EAPC = −2.45, 95% CI: −2.61 to −2.29) (Table 1).

### 3.2 National levels of gallbladder and biliary tract cancers burden

**Figure 1:**
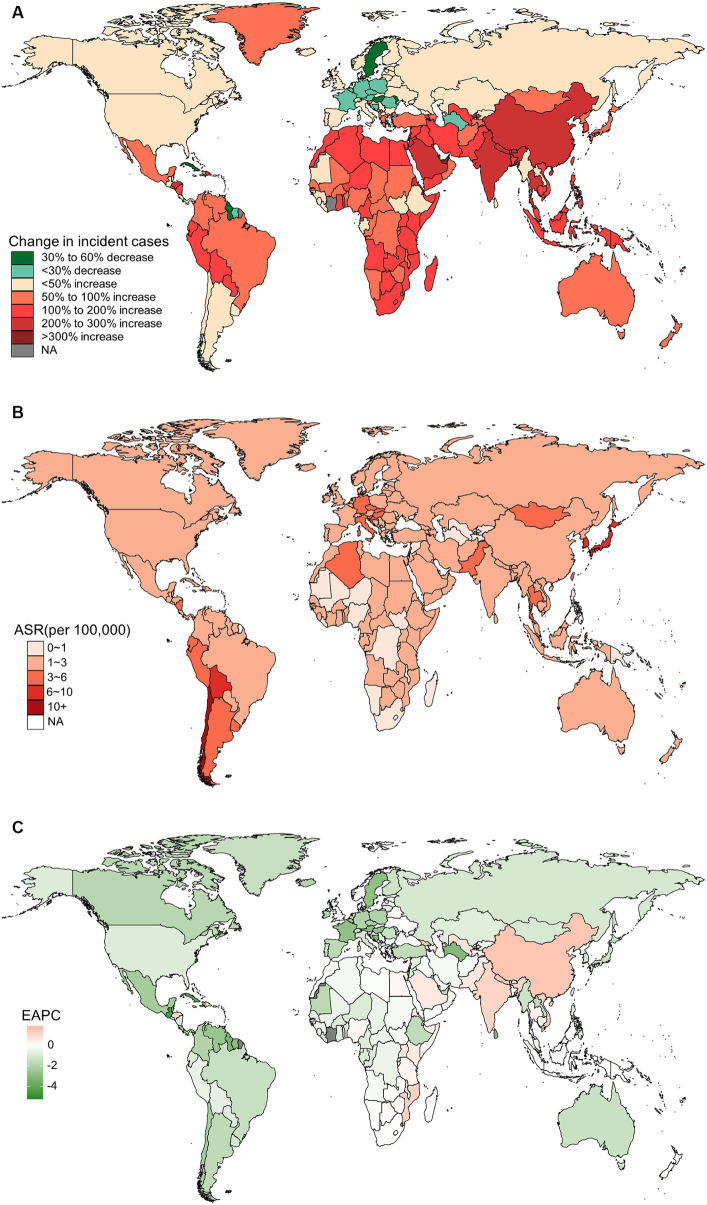
The global burden of incident gallbladder and biliary tract cancer (GBTC) for both sexes combined in 204 countries and territories. **(A)** The relative change in incident GBTC cases from 1990 to 2019. **(B)** The age-standardized incidence rate of GBTC in 2019. **(C)** The estimated annual percentage change of age-standardized incidence rate of GBTC from 1990 to 2019.

**Figure 2:**
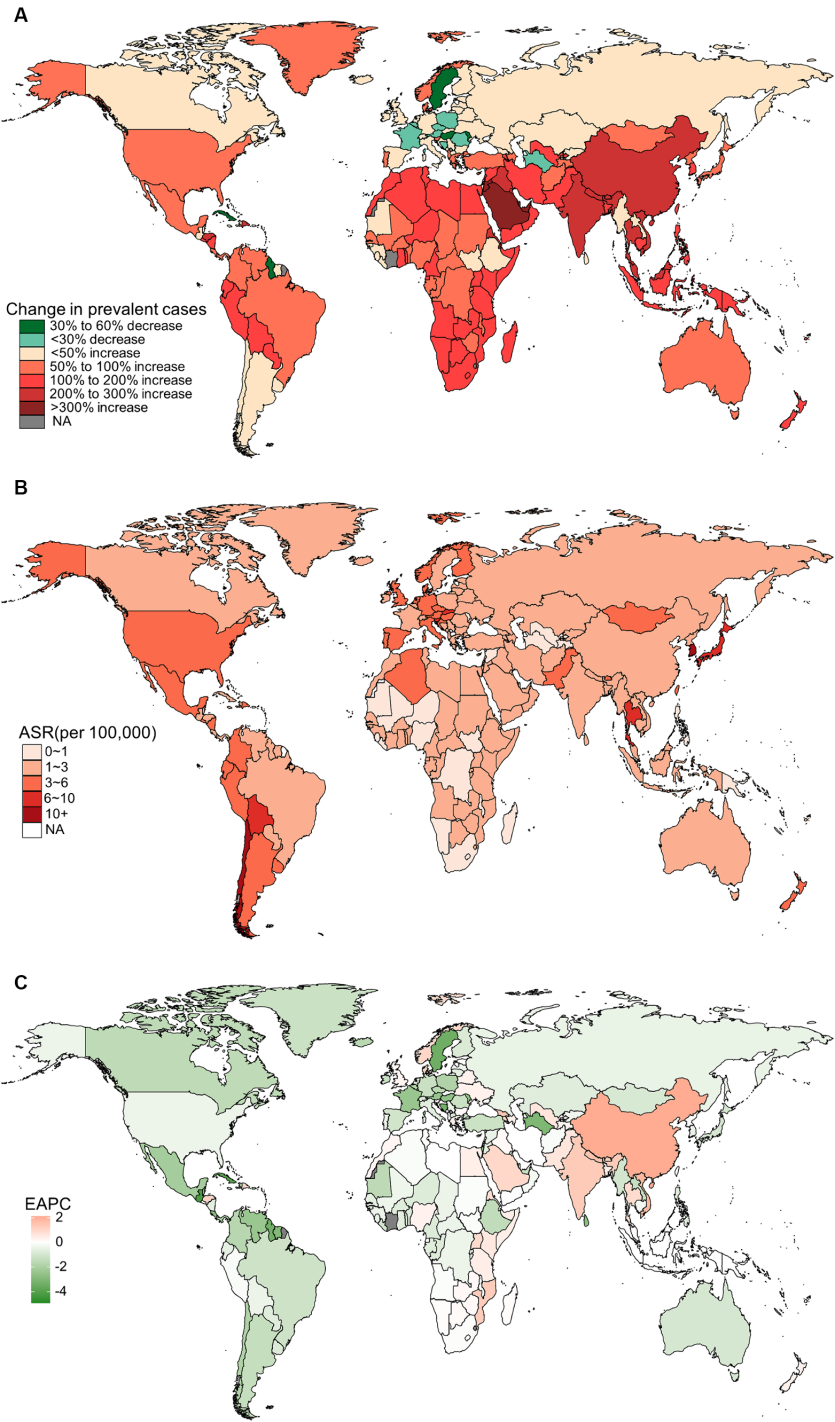
The global burden of prevalent gallbladder and biliary tract cancer (GBTC) for both sexes combined in 204 countries and territories. **(A)** The relative change in prevalent GBTC cases from 1990 to 2019. **(B)** The age-standardized prevalence rate of GBTC in 2019. **(C)** The estimated annual percentage change of age-standardized prevalence rate of GBTC from 1990 to 2019.

**Figure 3:**
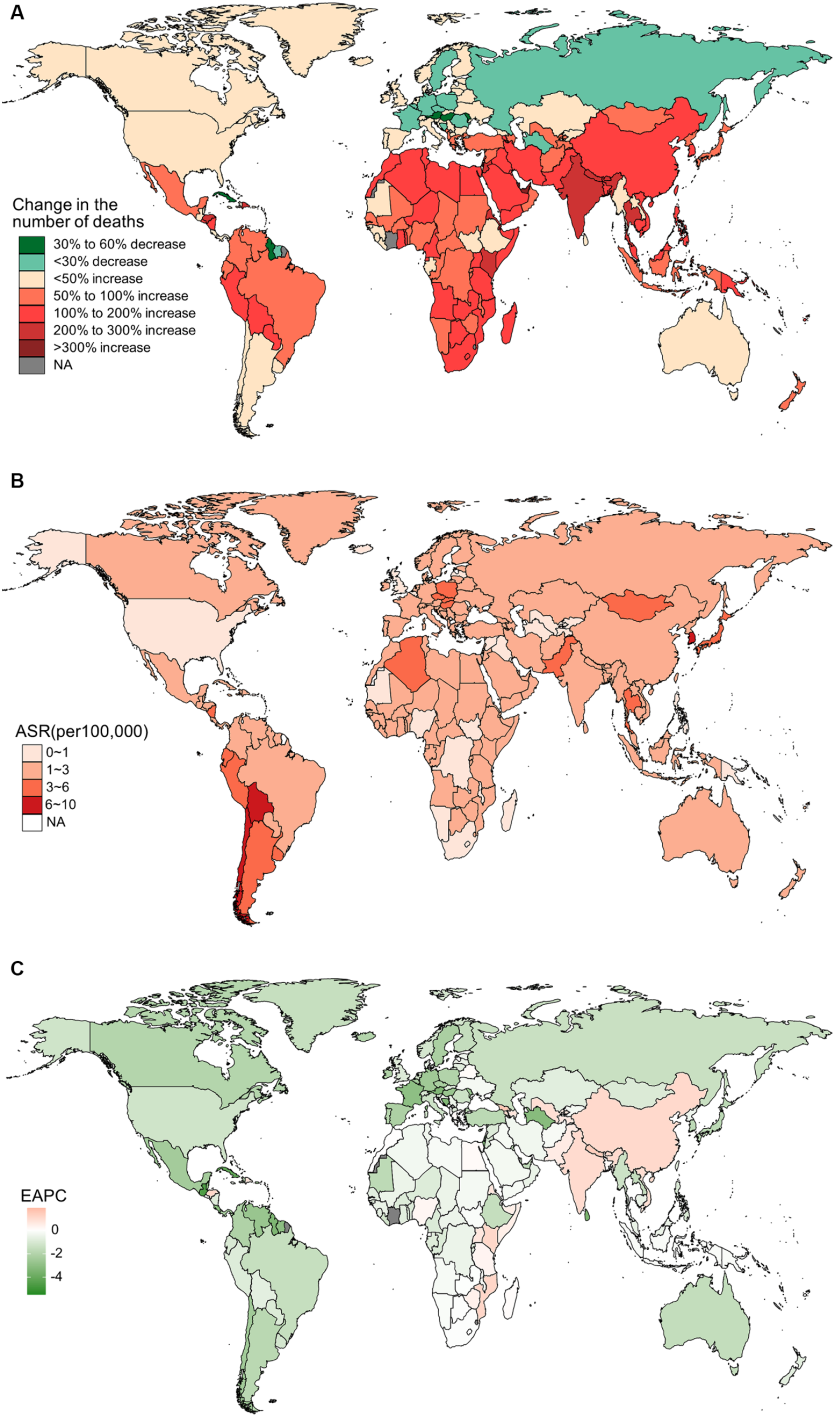
The distribution and trends of deaths caused by gallbladder and biliary tract cancer (GBTC) for both sexes combined in 204 countries and territories. **(A)** The relative change in the number of deaths caused by GBTC from 1990 to 2019. **(B)** The age-standardized mortality rate of GBTC in 2019. **(C)** The estimated annual percentage change of the age-standardized mortality rate of GBTC from 1990 to 2019.

**Figure 4:**
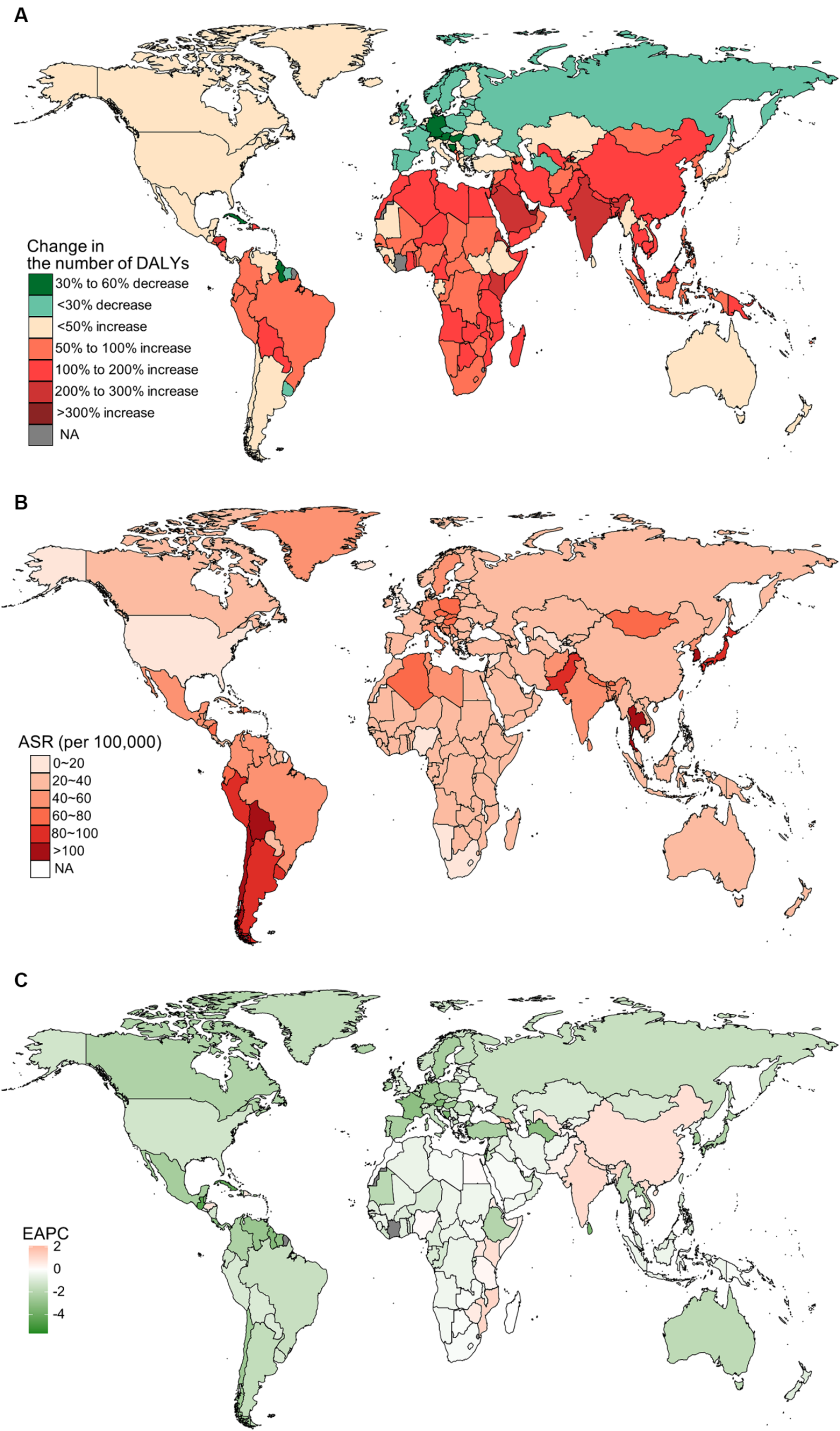
The distribution and trends of disability-adjusted life years (DALYs) caused by gallbladder and biliary tract cancer (GBTC) for both sexes combined in 204 countries and territories. **(A)** The relative change in the number of DALYs caused by GBTC from 1990 to 2019. **(B)** The age-standardized rate of DALYs of GBTC in 2019. **(C)** The estimated annual percentage change of the age-standardized rate of DALYs of GBTC from 1990 to 2019.

In 2019, China, India, and Japan had the highest absolute numbers of Gallbladder and biliary tract cancers burden around the world. From 1990 to 2019, the largest percentage increase was observed in the United Arab Emirates (Figures 1A–4A). In 2019, Chile had the highest age-standardized rates of incidence (11.1 per 10 ^5^, 95% UI: 8.5 to 15.2 per 10^5^), prevalence (14.0 per 10^5^, 95% UI: 10.6 to 18.9 per 10^5^), mortality (9.7 per 10^5^, 95% UI: 8.6 to 13.2 per 10^5^), and DALYs (204.3 per 105, 184.2 to 264.0 per 105) for gallbladder and biliary tract cancers (Figures 1B–4B). During 1990 to 2019, Lesotho showed the largest increase in age-standardized rates of incidence (EAPC = 1.90, 95% CI: 1.63 to 2.17), mortality (EAPC = 1.89, 95% CI: 1.62 to 2.17), and DALYs (EAPC = 2.09, 95% CI: 1.78 to 2.4), while China experienced the largest increase in age-standardized prevalence rate (EAPC = 2.12, 95% CI: 1.66 to 2.57) (Figures 1C–4C).

### 3.3 Gallbladder and biliary tract cancers burden by SDI quintile

In 2019, the high SDI quintile had the highest age-standardized rates of incidence, prevalence, and mortality for Gallbladder and biliary tract cancers burden; however, the low-middle SDI quintile reported the highest age-standardized DALYs rates (Supplementary Tables S1–S4). Between 1990 and 2019, the age-standardized incidence, prevalence, and mortality rates only decreased in the high and high-middle SDI quintiles (Table 1). However, high, high-middle, and middle SDI quintiles reported a decreasing trend in the age-standardized rate of DALYs (Supplementary Tables S1–S4).

### 3.4 Age and sex patterns of gallbladder and biliary tract cancers burden

**Figure 5:**
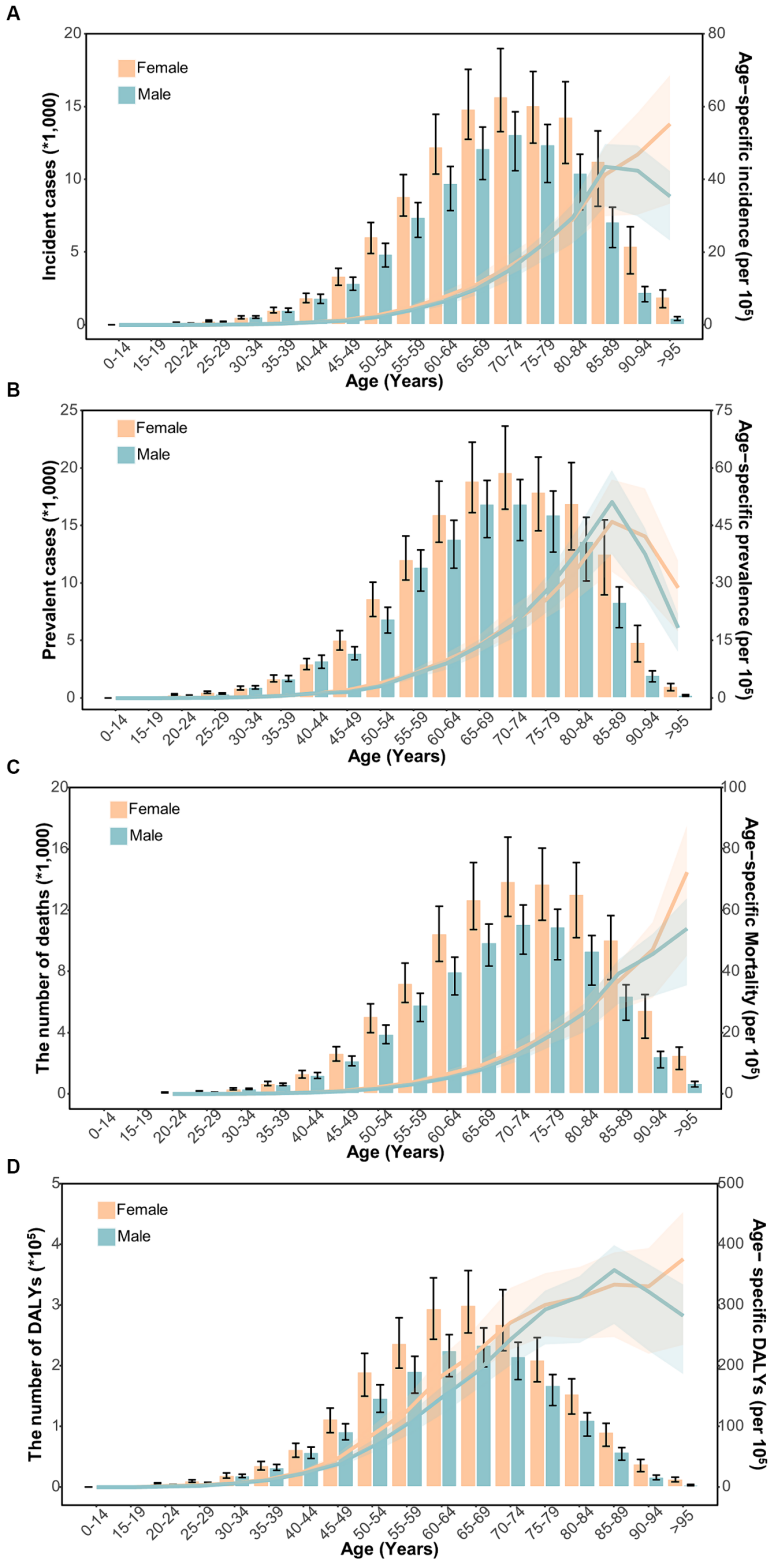
The distribution and trend of incidence **(A)**, prevalence **(B)**, mortality **(C)**, and DALYs **(D)** due to gallbladder and biliary tract cancer in 2019 by age and sex.

**Figure 6:**
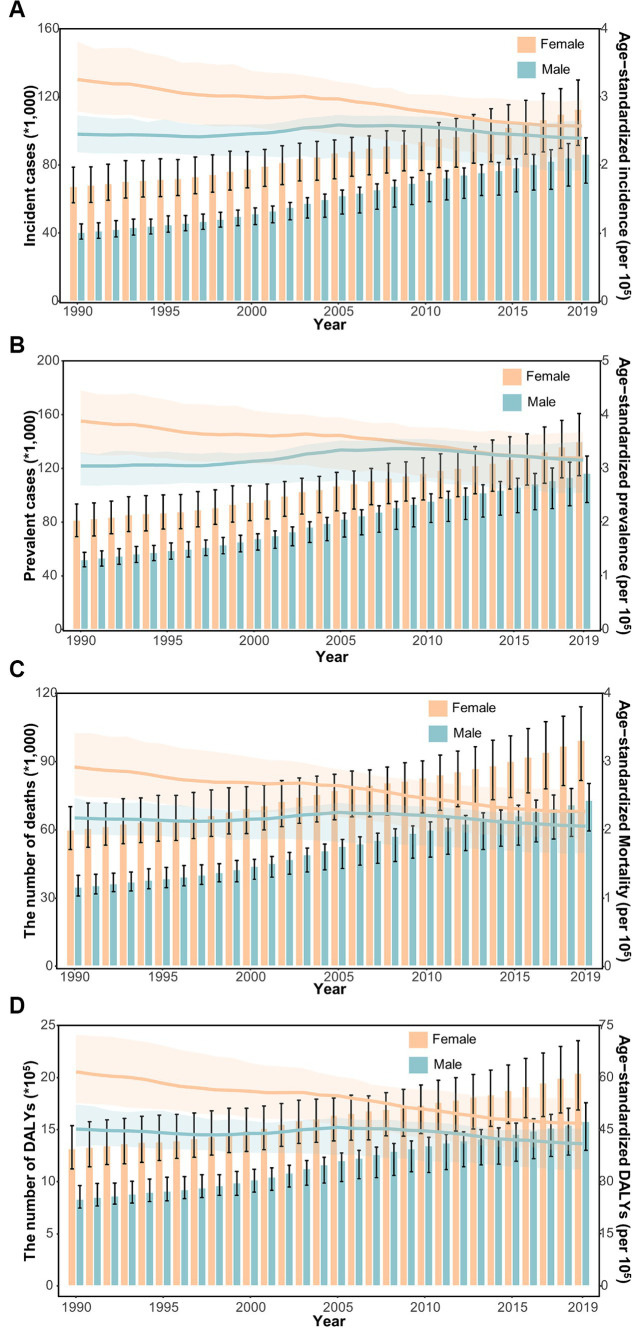
The distribution and trend of incidence **(A)**, prevalence **(B)**, mortality **(C)**, and DALYs **(D)** due to gallbladder and biliary tract cancer from 1990 to 2019 by sex.

**Figure 7:**
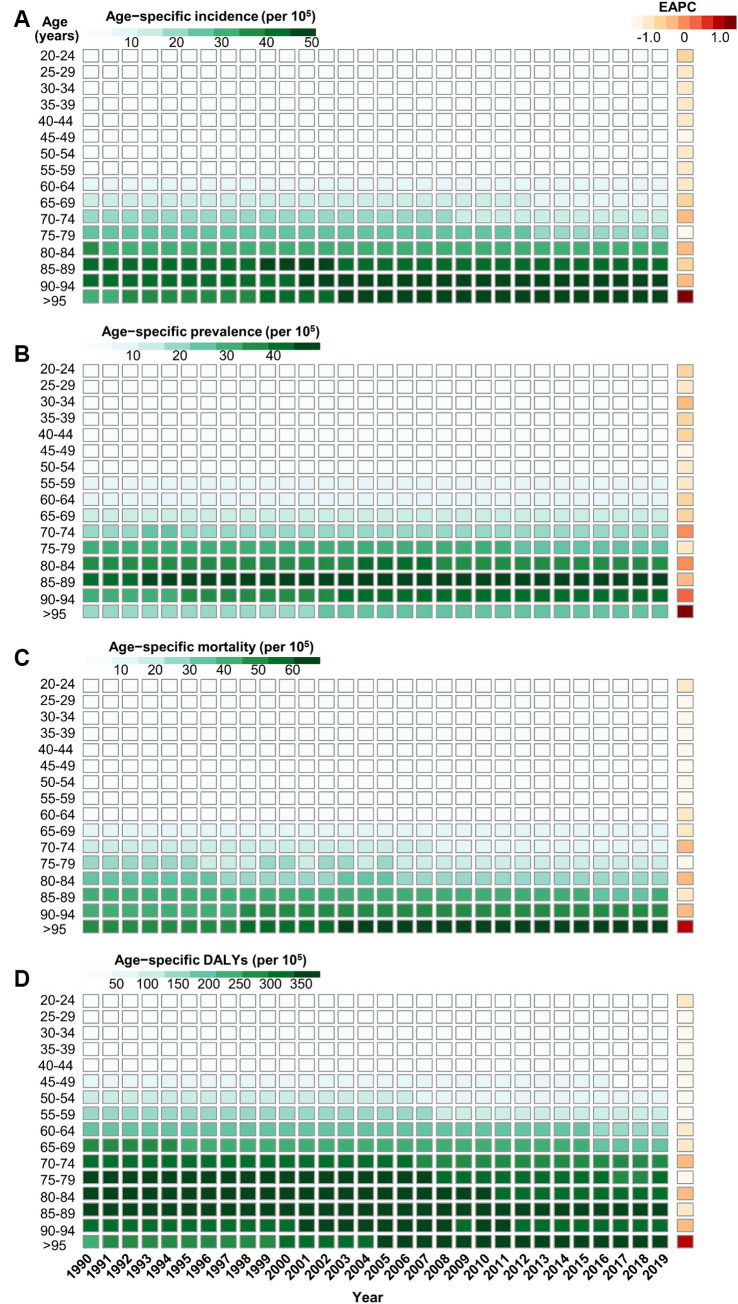
The trend of age-specific rates of incidence **(A)**, prevalence **(B)**, mortality **(C)**, and DALYs **(D)** due to gallbladder and biliary tract cancer by age from 1990 to 2019.

In general, the age-specific rates of incidence, prevalence, and mortality, and DALYs for gallbladder and biliary tract cancers tended to increase with age up until around 80 years old (Figure 5). The age-specific rates of gallbladder and biliary tract cancers burden were generally higher among females than males across most age groups (Figure 5). From 1990 to 2019, the age-standardized incidence and prevalence rates increased among males but decreased among females (Figures 6A,B). Moreover, females experienced a greater decrease in age-standardized mortality and DALYs rates compared to males (Figures 6C,D). The most significant decrease in age-specific rates of incidence, prevalence, mortality, and DALYs were observed in individuals aged 45–49 years (Figure 7). Notably, the global number of incident cases of early-onset gallbladder and biliary tract cancers increased by 52.4% from 1990 to 2019, with the greatest increase detected in low SDI areas (Table 2).

**Table 2:** The percentage change of early- and later-onset GBTC cases from 1990 to 2019 by SDI and sex.

Socio-demographic index (SDI)	Incidence		Prevalence		Deaths		DALYs	
	Number in 2019 × 10^3^	Percentage change in absolute number (%)	Number in 2019 × 10^3^	Percentage change in absolute number (%)	Number in 2019 × 10^3^	Percentage change in Absolute number (%)	Number in 2019 × 10^3^	Percentage change in absolute number (%)
*Sex = Both*								
Global								
Early-onset	13.72	52.36	21.77	58.55	9.76	46.47	456.17	44.45
Later-onset	185.49	87.78	234.57	95.49	162.68	84.46	3165.31	72.24
High SDI								
Early-onset	1.90	−18.86	4.06	−8.87	0.95	−30.38	43.93	−30.55
Later-onset	61.94	45.38	90.56	57.31	47.46	38.64	743.87	14.82
High-middle SDI								
Early-onset	3.19	34.16	5.24	48.64	2.17	21.25	100.91	18.90
Later-onset	47.59	72.86	59.44	85.98	41.87	64.39	819.11	53.29
Middle SDI								
Early-onset	3.19	34.16	6.37	94.42	3.03	57.57	141.47	53.16
Later-onset	4.17	74.05	47.83	179.31	39.27	149.34	835.07	135.05
Low-middle								
Early-onset	3.29	132.86	4.53	143.58	2.64	124.23	123.89	121.84
Later-onset	26.32	177.57	28.29	183.47	26.05	175.11	582.98	162.60
Low SDI								
Early-onset	1.17	144.89	1.56	155.74	0.97	137.16	45.75	137.27
Later-onset	7.94	147.91	8.37	153.34	7.97	146.77	183.02	136.62
*Sex = Female*								
High SDI								
Early-onset	0.85	−24.44	1.85	−13.85	0.44	−35.47	20.38	−35.59
Later-onset	33.07	26.12	45.56	33.61	26.01	21.15	382.18	−1.45
High-middle SDI								
Early-onset	1.47	12.85	2.49	26.73	1.01	1.82	46.55	−0.02
Later-onset	27.11	47.15	33.00	56.57	24.08	40.40	456.07	30.06
Middle SDI								
Early-onset	2.02	52.80	3.15	69.14	1.50	40.38	69.60	36.47
Later-onset	23.44	136.71	26.56	151.26	22.18	126.32	463.63	112.34
Low-middle								
Early-onset	2.07	130.98	2.89	139.62	1.68	125.34	79.11	122.72
Later-onset	16.85	184.93	17.97	189.62	16.71	183.80	374.76	171.66
Low SDI								
Early-onset	0.77	145.40	1.05	156.62	0.65	138.75	30.57	139.21
Later-onset	5.13	166.56	5.38	171.38	5.15	167.03	118.82	155.48
*Sex = Male*								
High SDI								
Early-onset	1.05	−13.53	2.21	−4.25	0.51	−25.40	23.55	−25.49
Later-onset	28.87	76.21	45.00	91.73	21.45	68.03	361.69	39.10
High-middle SDI								
Early-onset	1.72	59.91	2.75	76.30	1.16	45.00	54.36	41.88
Later-onset	20.48	124.91	26.44	142.93	17.79	113.92	363.04	97.64
Middle SDI								
Early-onset	2.15	100.37	3.23	127.66	1.54	79.11	71.86	73.73
Later-onset	18.20	202.69	21.28	224.61	17.08	187.27	371.44	171.26
Low-middle								
Early-onset	1.22	135.38	1.65	150.99	0.96	122.33	44.79	120.27
Later-onset	9.46	165.47	10.32	173.49	9.33	160.70	208.22	147.74
Low SDI								
Early-onset	0.40	141.21	0.51	156.50	0.32	130.71	15.18	133.39
Later-onset	2.81	119.98	2.997	126.53	2.82	117.32	64.20	108.18

### 3.5 The influential factors for EAPC

**Figure 8:**
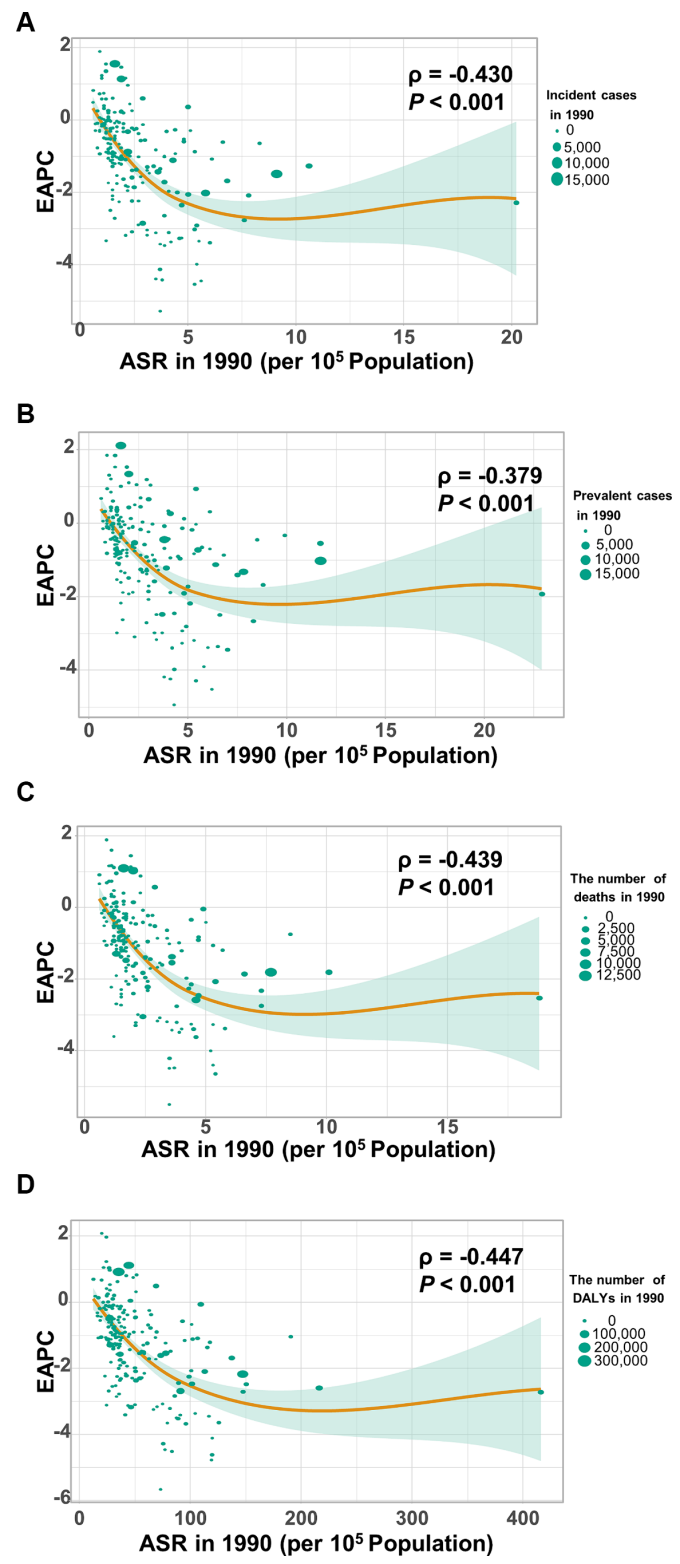
The correlation between estimated annual percentage change (EAPC) and age-standardized rate of gallbladder and biliary tract cancer (GBTC) in 1990. **(A)** The EAPC negatively associated with the age-standardized incidence. **(B)** The EAPC negatively associated with the age-standardized prevalence. **(C)** The EAPC negatively associated with the age-standardized mortality. **(D)** The EAPC negatively associated with the age-standardized rate of DALYs due to GBTC. The circles represent countries. The size of each circle is proportional to the number of incident cases, prevalent cases, deaths, and DALYs, respectively. The ρ indices and *p* values were derived from Pearson correlation analysis.

**Figure 9:**
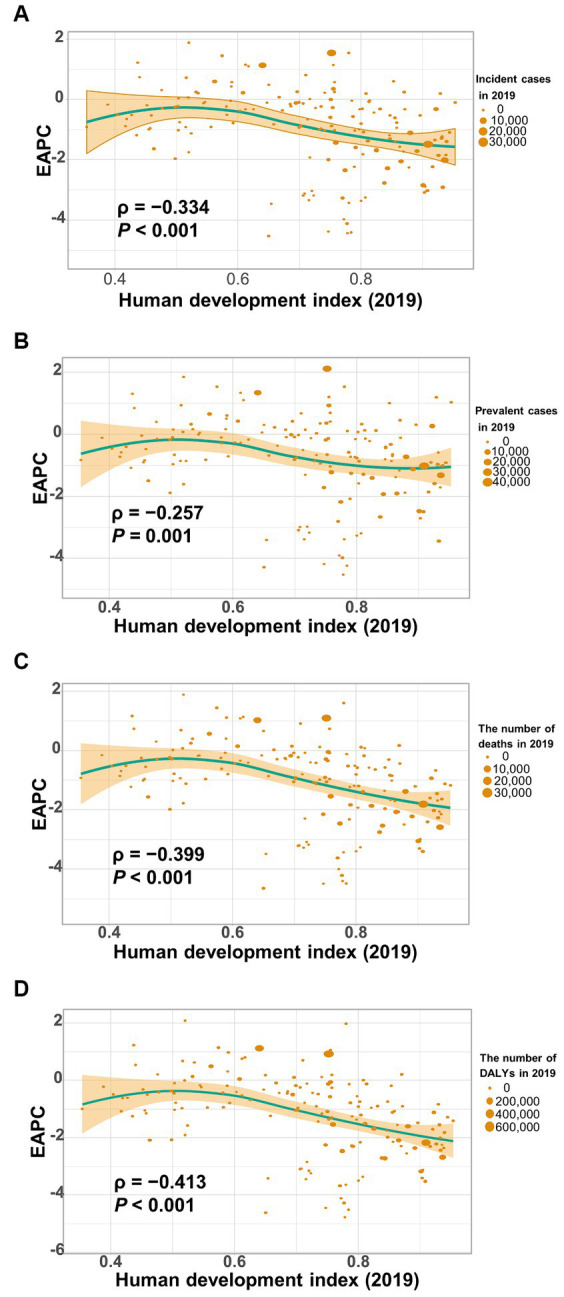
The correlation between estimated annual percentage change (EAPC) of age-standardized rate of gallbladder and biliary tract cancer (GBTC) and human development index (HDI) in 2019. **(A)** The EAPC of age-standardized incidence negatively associated with the HDI. **(B)** The EAPC of age-standardized prevalence negatively associated with the HDI. **(C)** The EAPC of the age-standardized mortality negatively associated with the HDI. **(D)** The EAPC of the age-standardized rate of DALYs negatively associated with the HDI. The circles represent countries that were available on HDI data. The size of each circle is proportional to the number of incident cases **(A)**, prevalent cases **(B)**, deaths **(C)**, and DALYs **(D)**, respectively. The ρ indices and p values were derived from Pearson correlation analysis.

Figure 8 shows a significant negative association (*ρ* = −0.379 to −0.447, *p* < 0.001) between the estimated annual percent change and the age-standardized rates of incidence (Figure 8A), prevalence (Figure 8B), mortality (Figure 8C), and DALYs (Figure 8D) for gallbladder and biliary tract cancer in 1990. These rates in 1990 represent the initial disease burden caused by gallbladder and biliary tract cancer during the study period. Additionally, a significant negative association (*ρ* = −0.257 to −0.413, *p* < 0.05) was observed between the human development index (HDI) in 2019 and the EAPC of the age-standardized rates of incidence (Figure 9A), prevalence (Figure 9B), mortality (Figure 9C), and DALYs (Figure 9D). The HDI score in 2019 serves as an indicator of economic development and healthcare accessibility in each country. Our findings demonstrate that countries with higher HDI scores have experienced a slower increase or a more rapid decrease in the age-standardized rates of incidence, prevalence, mortality, and DALYs for gallbladder and biliary tract cancer from 1990 to 2019 (Figure 9). Conversely, countries with higher HDI scores have significantly higher age-standardized rates of incidence, prevalence, and mortality (Supplementary Figures S1A–C). Importantly, the age-standardized rate of DALYs attributed to gallbladder and biliary tract cancer is positively associated with the HDI score when the HDI score is below 0.82 (Supplementary Figures S1D). However, a significant negative association is detected when the HDI score is above 0.82.

### 3.6 Risk factors for gallbladder and biliary tract cancers

Globally, a considerable fraction of deaths and DALYs from gallbladder and biliary tract cancers were attributable to a high BMI (> 23.0 kg/m^2^) available in the GBD study 2019 (Supplementary Figures S2). In 1990, 13.4 and 13.2% of deaths and DALYs from these cancers were associated with a high BMI, which increased to 15.2 and 15.7% in 2019, respectively (Supplementary Figures S2). More importantly, the impact of the risk factor varied by region, with the greatest impact seen in Central Europe in 1990 (24.5% of deaths and 24.7% of DALYs attributable to a high BMI) and in Eastern Europe in 2019 (28.4% of deaths and 28.2% of DALYs attributable to a high BMI), for both sexes combined. Conversely, the lowest proportion of age-standardized deaths and DALYs due to a high BMI in 1990 and in 2019 were observed in South Asia (4.9% of deaths and 5.1% of DALYs attributable to a high BMI) and High-income Asia Pacific (8.3% of deaths and 8.5% of DALYs attributable to a high BMI), respectively.

**Figure 10:**
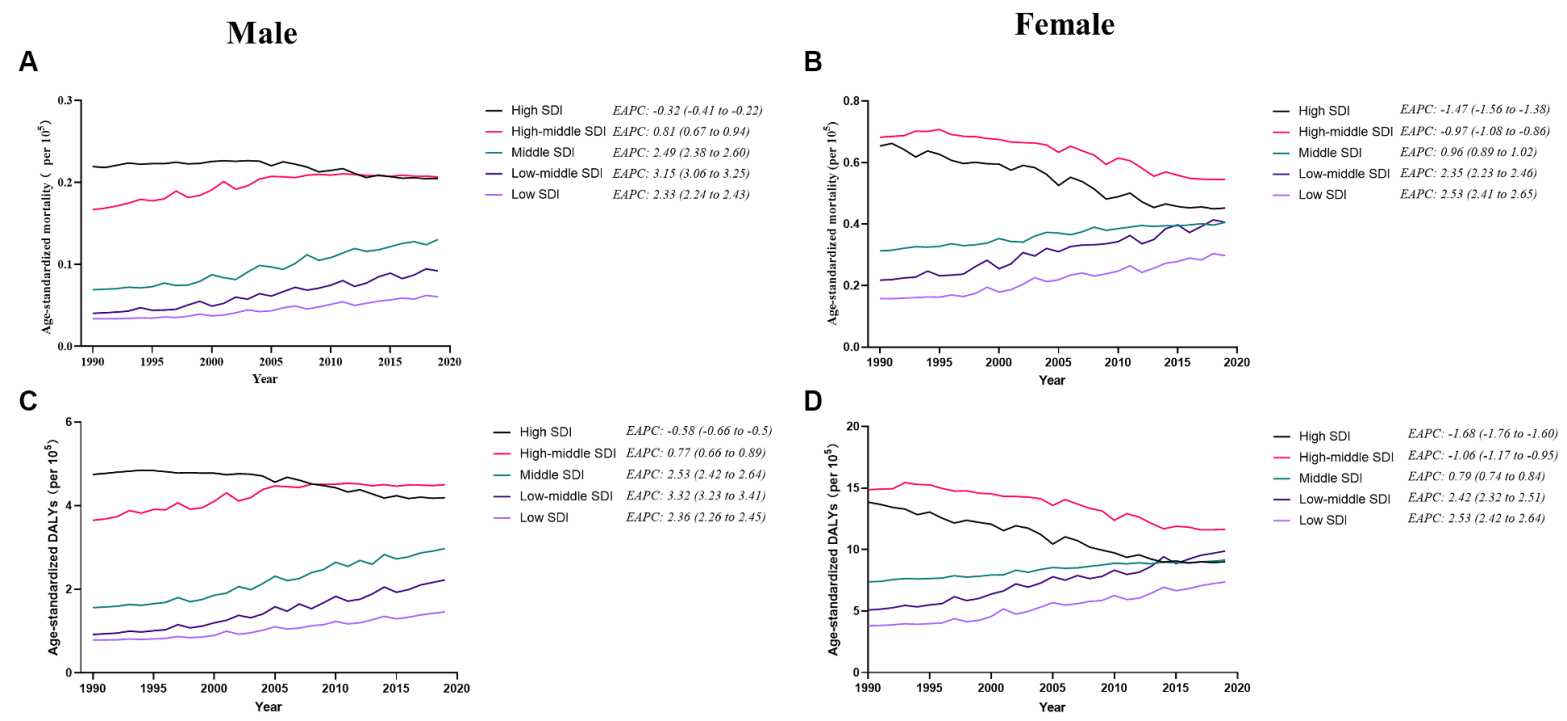
The temporal trend of high BMI-attributable mortality **(A,B)** and DALYs **(C,D)** of gallbladder and biliary tract cancer, categorized by sex and SDI areas.

Furthermore, during 1990 to 2019, the temporal trend in risk factor-attributable deaths and DALYs for gallbladder and biliary tract cancers varied across SDI areas and by sex (Figure 10). Among males, the age-standardized rate of mortality and DALYs was on the rise in all SDI areas, except for high SDI areas, with the most significant increase in low-middle SDI areas (Figures 10A,C). Additionally, deaths and DALYs caused by gallbladder and biliary tract cancers in females associated with a high BMI tend to trend downward in both high and high-middle SDI areas, while increasing in the remaining 3 SDI areas, particularly in low SDI areas (Figures 10B,D).

### 3.7 Prediction of age-standardized rate of incidence and mortality

**Figure 11:**
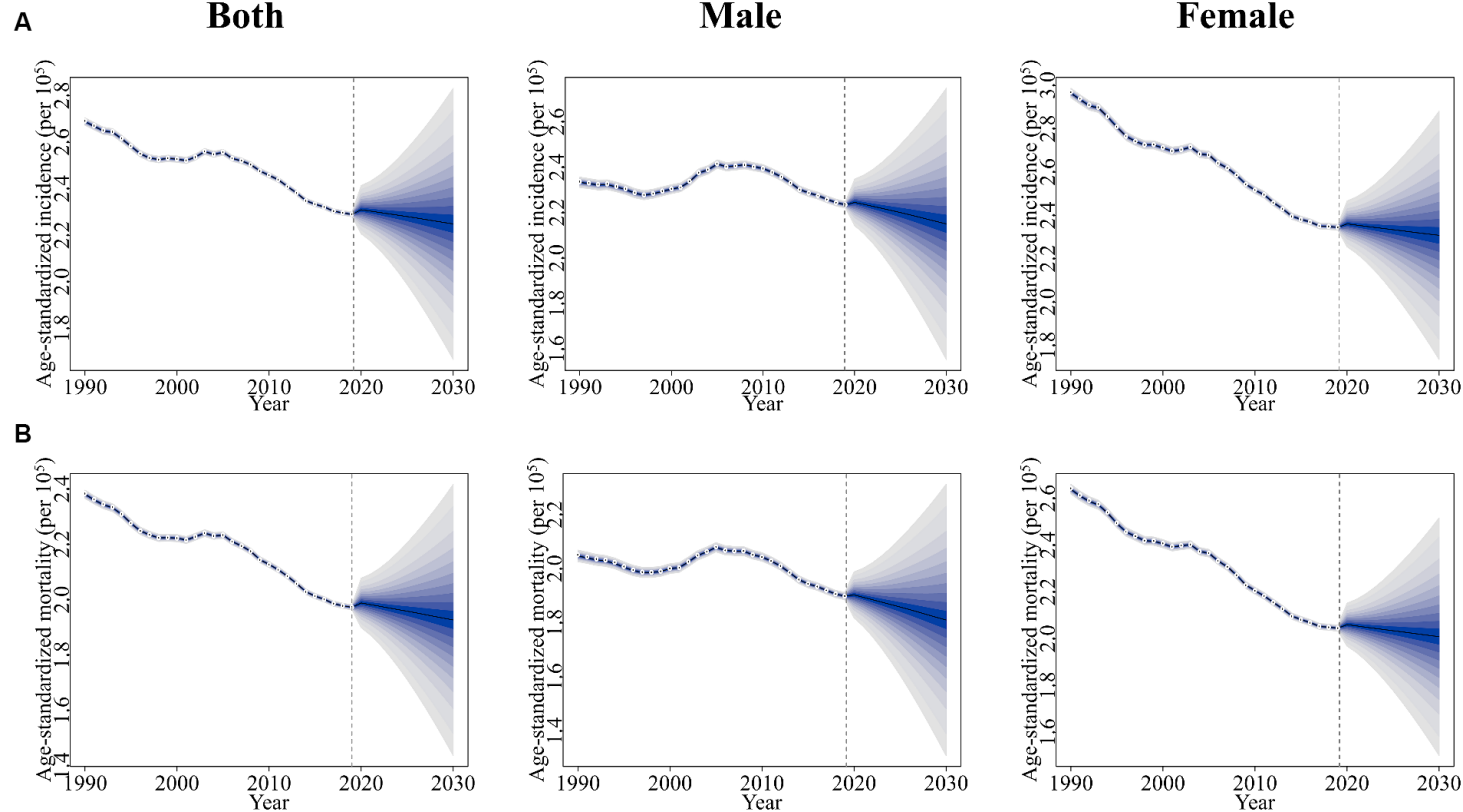
Trends of age-standardized incidence **(A)** and age-standardized mortality **(B)** of gallbladder and biliary tract cancers for both sexes combined (left), male (middle), and female (right): observed rates (1990–2019) and predicted rates (2020–2030). The blue region in shows the upper and lower limits of the 95% UI.

The prediction and the changing trends of age-standardized incidence rate and age-standardized mortality rate of gallbladder and biliary tract cancers were shown in Figure 11. In general, the age-standardized rate of incidence and mortality of gallbladder and biliary tract cancers would continue to decrease globally from 2020 to 2030, with higher rates in women than in men. In 2030, the globally projected age-standardized incidence rate of gallbladder and biliary tract cancers is 2.25 (1.67 to 2.83) per 10^5^ for both sexes combined, 2.15 (1.55 to 2.75) per 10^5^ for males, and 2.31 (1.74 to 2.88) per 10^5^ for females (Figure 11A). Additionally, the globally projected age-standardized mortality rate of gallbladder and biliary tract cancers is 1.93 (1.44 to 2.42) per 10^5^ for both sexes combined, 1.81 (1.31 to 2.31) per 10^5^ for males, and 2.01 (1.5 to 2.52) per 10^5^ for females (Figure 11B).

## 4 Discussion

Based on the GBD study 2019, this analysis provides an updated and comprehensive assessment of the disease burden attributed to gallbladder and biliary tract cancer at the global, regional, and national level in 2019, as well as the temporal trends over the past 30 years. From 1990 to 2019, the number of incident cases, prevalent cases, deaths, and DALYs significantly increased worldwide by 1.85-fold, 1.92-fold, 1.82-fold, and 1.68-fold, respectively. These trends reflect changes in population structure (aging and growth) and the global distribution of risk factors for gallbladder and biliary tract cancer (21, 22). However, the age-standardized rates of incidence, prevalence, mortality, and DALYs tend to trend downward globally over time (Table 1). It is worth noting that higher socio-demographic index was associated with a lesser increase in the absolute number of burdens caused by gallbladder and biliary tract cancer. Additionally, decreasing trends in the age-standardized rates of incidence, prevalence, mortality, and DALYs were observed with increasing SDI or HDI score (Table 1; Figure 9), which may be attributed to the implementation of routine cholecystectomy in high-income countries (9). Unhealthy lifestyle and environmental risk factors contribute to a significant proportion of new cancer cases in high-income countries, highlighting the importance of socio-economic development and early prevention in controlling gallbladder and biliary tract cancer (21). Because gallbladder and biliary tract cancers are highly fatal disease with a high mortality-to-incidence ratios, YLLs account for almost all DALYs (98.7%). However, YLLs have shown a more rapid decrease than YLDs (Supplementary Tables S5, S6), indicating advancements in awareness, early screening, diagnostics, and therapies (23, 24).

The disease burden of gallbladder and biliary tract cancer varies substantially by geographic region, national level, age, and sex. The largest increase in the number of and age-standardized rates of burden were observed in East Asia and South Asia. Different geographical risk factors, distinct subtypes of gallbladder and biliary tract cancer, and potential genetic predispositions or ethnicity likely contribute to the varied disease burden patterns between geographic regions (24, 25). According to anatomical location, gallbladder and biliary tract cancer can be categorized into intrahepatic, perihilar, distal cholangiocarcinoma, and gallbladder cancer, each with varying incidence, clinical presentation, natural history, molecular profile, and prognosis (9, 26). Many studies have shown a rising incidence of intrahepatic cholangiocarcinoma worldwide, particularly in high-income Western countries, while the incidence of extrahepatic cholangiocarcinoma and gallbladder cancer has remained stable over the past decades (27, 28). However, extrahepatic cholangiocarcinoma continues to be more common than intrahepatic cholangiocarcinoma in Sweden and the United States (27, 29), and the incidence of cholangiocarcinoma is approximately 40 times higher in East Asia (China) or Southeast Asia (Thailand) compared to Western countries (30, 31). It is worth noting that parasitic infections and hepatolithiasis are more prevalent in Asia and are strong risk factors for cholangiocarcinoma, particularly intrahepatic cholangiocarcinoma (32). Moreover, although most Asian nations have seen a decline in the prevalence of Hepatitis B virus (HBV) infection due to universal HBV vaccination, the prevalence of HBV infection exceeding 8% is mainly concentrated in Asia and Africa, which account for nearly 70% of all HBV-infected individuals worldwide. In contrast, Western countries have a low prevalence of HBV infection below 2% (33, 34). Chronic HBV infection is a significant risk factor not only for cirrhosis/hepatocellular carcinoma but also for gallbladder and biliary tract cancer (32, 35, 36). Furthermore, host genetic factors may contribute to different susceptibility to gallbladder and biliary tract cancer among ethnic populations (37). A previous study has reported that Asian-Americans have a higher incidence of biliary tract cancer than the general US population, suggesting that the higher burden associated with biliary tract cancer in the Asian population cannot be solely attributed to ecological and geographical factors (38). Additionally, it has been found that the incidence of intrahepatic cholangiocarcinoma is 2 times higher, with an inferior 5-year survival rate, in Hispanic Americans compared to non-Hispanic individuals (39, 40). By the way, 26.6% of Hispanic Americans lived in poverty and 30.7% were uninsured (41), and the higher mortality rates of cholangiocarcinoma among them were associated with both genetic factors and poverty (25). The highest age-standardized incidence rate was observed in Southern Latin America (Chile, Bolivia, Uruguay, Argentina, and Peru), where the most frequently diagnosed anatomic subtype was gallbladder cancer, which is likely due to the high prevalence of cholelithiasis (5, 36, 42–45). In Chile, the prevalence of gallstone disease in female adults was over twice as high as in male adults (45% vs. 20%) (46). Coincidentally, the age-standardized incidence rate of gallbladder and biliary tract cancer was also almost 2 times higher in females than in males (14.0 per 10^5^ persons vs. 7.5 per 10^5^ persons in 2019), and gallbladder cancer affected females more frequently than males (45).

Previous research has revealed that having gallstones is the primary risk factor for gallbladder and biliary tract cancers; the incidence rates of gallbladder and biliary tract cancers was strongly associated with the global prevalence of gallstone. High or medium prevalence of gallstones was observed in regions of American, Europe, and Australia, while countries in Africa and Eastern Asia have low risk for gallstones (11). Other risk factors include obesity, diabetes, dietary risk factors, and chronic infections. The prevalence of obesity was historically lower in Eastern countries than in Western countries; however, faster increasing trend in obesity prevalence was reported in Eastern countries because unhealthy lifestyles had shifted from West countries to East countries which underwent a dietary transition toward western diets (47). Therefore, policymakers must be proactively aware of these shifts and established evidence-based interventions to address this epidemic. Moreover, chronic infection due to HBV, parasite, and Aspergillus flavus were predominant in regions of Asia (34, 48). Therefore, preventing and controlling the numerous pathogens driving gallbladder and biliary tract cancers in Eastern countries is a public health priority.

In addition to metabolic factors, environmental exposure and genetic susceptibility, socioeconomic status may also contribute to the regional variation in the burden of gallbladder and biliary tract cancer. Generally, lower socioeconomic status is associated with a higher incidence of cancer and poorer prognosis (49–51). However, both previous studies and our own research have shown that the burden of gallbladder and biliary tract cancer increases with the SDI score (Supplementary Figure S1) (5). This paradox can be partially explained by limited access to screening and diagnosis, as well as inadequate cancer registries and reporting systems in underdeveloped countries, leading to under-diagnosis or under-reporting biases (21). On the other hand, areas with low SDI scores have shown an upward trend in the age-standardized rate of gallbladder and biliary tract cancer burden, which may be attributed to the increasing implementation of routine screening and early diagnosis (15). Additionally, the adoption of a westernized diet and lifestyle in underdeveloped countries, along with the associated increase in overweight and diabetes, could also contribute to the rising burden of gallbladder and biliary tract cancer (52, 53). Just as shown in our results, high BMI-attributable mortality and DALYs of gallbladder and biliary tract cancer have declined over the past 30 years in high or high-middle SDI areas, while they have been on the rise in areas with a lower SDI score (Figure 10). Fortunately, most countries have shown a decreasing trend in the age-standardized rates of gallbladder and biliary tract cancer burden, with the most significant decrease observed in Central Latin America or the Caribbean (Figures 1C, 2C, 3C, 4C). Notably, a significant proportion of deaths and DALYs from gallbladder and biliary tract cancers worldwide can be attributed to a high BMI, however, the ranking of the risk factor-attributable proportion among the 21 GBD regions have changed from 1990 to 2019. For example, in the Asia region, in 1990, the proportion of high BMI-associated deaths or DALYs was significant higher in High-income Asia Pacific than Southeast Asia, South Asia, and East Asia. However, by 2,109, the High-income Asia Pacific ranked behind Southeast Asia, South Asia, and East Asia. In the recent decades, due in part to rapid nutrition and lifestyle transitions, obesity prevalence had surged in Asia, with the exception of Japan where the increase has been significantly slower (54, 55).

In general, males have a higher risk for most cancer types compared to females (56, 57). For example, in the United Kingdom, males have a 1.6 times higher incidence rate and a 1.7 times higher mortality rate for the most common cancers (58). However, both the elderly and females are more susceptible to gallbladder and biliary tract cancer (Figures 5–7). The mechanisms underlying these sex differences are multifaceted and include factors such as epigenetics, genetics, endocrine factors, and behavior (59). It has been found that being female is a significant risk factor for cholelithiasis (44, 60). Moreover, worldwide, the crude mortality rate of cholangiocarcinoma is higher in men than in women (24). Furthermore, the increasing burden of early-onset gallbladder and biliary tract cancer is a cause for concern, especially in lower SDI areas and among males (Table 2; Supplementary Tables S8–S10). Several studies have highlighted the rising trend in the burden of biliary tract cancer among younger adults in different geographic regions (29, 61, 62).

Previous studies have outlined the global burden and trend of gallbladder and biliary tract cancer from 1990 to 2017, based on the GBD Study 2017 (5, 63, 64). However, as each new version of the GBD is released, data is revised and new methodologies are applied, leading to updated estimations for the entire time period that surpass previously disclosed GBD round estimations (13). This current study presents a systematic and up-to-date assessment of the disease burden resulting from gallbladder and biliary tract cancers on a global, regional, and national scale, utilizing the GBD Study 2019. Not only does this encompass data for 2018 and 2019, but it also expands its survey coverage from 195 to 204 countries or territories. In comparison to prior GBD studies, GBD study 2019 has integrated new systematic reviews, cohorts, trials, and case–control studies. Furthermore, the Bayesian age-period-cohort model was utilized in this report to predict the age-standardized rate of incidence and mortality for gallbladder and biliary tract cancers from 2020 to 2030.

There are some limitations in the current study. Firstly, we failed to evaluate the distribution and trend of disease burden for gallbladder and biliary tract cancer based on anatomical subsite. The changing disease burden patterns of gallbladder and biliary tract cancer may be influenced by the change in anatomical subtype profile. Secondly, different gallbladder and biliary tract subtypes have varying risk factors, molecular characteristics, and prognosis. Therefore, pooling the gallbladder and biliary tract burden may mask the changing etiology and compromise cancer control efforts, despite improving statistical power (65). Thirdly, the accuracy and robustness of the GBD study estimates depend on the quality and quantity of the original data or publications, which may introduce potential bias due to changes in ICD-classification or coding misclassification (66).

## 5 Conclusion

Our study provides comprehensive insights into the distribution and dynamic trends of gallbladder and biliary tract burden over the past three decades, from multiple perspectives. These findings emphasize the importance of promoting a healthy lifestyle as a population-level cancer prevention strategy and tailoring cancer control actions based on localized risk factors and the epidemic profiles of gallbladder and biliary tract cancer by anatomical subtype.

## Data availability statement

The datasets presented in this study can be found in online repositories. The names of the repository/repositories and accession number(s) can be found in the article/Supplementary material.

## Author contributions

JS: Data curation, Formal analysis, Investigation, Methodology, Writing – original draft, Writing – review & editing. YL: Conceptualization, Data curation, Formal analysis, Investigation, Methodology, Visualization, Writing – original draft, Writing – review & editing. XH: Conceptualization, Project administration, Supervision, Writing – original draft, Writing – review & editing.

## Footnotes

^1^ http://www.healthdata.org/gbd/about/protocol

^2^ http://ghdx.healthdata.org/gbd-results-tool

^3^ http://hdr.undp.org/en/data

^4^ https://seer.cancer.gov/stdpopulations/world.who.html

^5^ https://population.un.org/wpp/Download/Standard/CSV/
