# Supplementary material for: Global, regional, and national burden and trends analysis of gallbladder and biliary tract cancer from 1990 to 2019 and predictions to 2030: a systematic analysis for the Global Burden of Disease Study 2019
Source: Front Med (Lausanne). 2024 Apr 4;11:1384314. doi: 10.3389/fmed.2024.1384314 (PMC11024434; doi:10.3389/fmed.2024.1384314)

Supplementary materials

Supplementary Table S1. The incident cases and age-standardized incidence of GBTC in 1990 and 2019, and its temporal trends from 1990 to 2019

Supplementary Table S2. The prevalent cases and age-standardized prevalence of GBTC in 1990 and 2019, and its temporal trends from 1990 to 2019

Supplementary Table S3. The number of death and age-standardized mortality rate of GBTC in 1990 and 2019, and its temporal trends from 1990 to 2019

Supplementary Table S4. The number and age-standardized rate of DALYs due to GBTC in 1990 and 2019, and its temporal trends from 1990 to 2019

Supplementary Table S5. The number and age-standardized rate of YLDs due to GBTC in 1990 and 2019, and its temporal trends from 1990 to 2019

Supplementary Table S6. The number and age-standardized rate of YLLs due to GBTC in 1990 and 2019, and its temporal trends from 1990 to 2019

Supplementary Table S7. The change of GBTC cases between 1990 and 2019 at national level,

both sexes

Supplementary Table S8. The percentage change of GBTC cases from 1990 to 2019 by SDI and age

Supplementary Table S9. The percentage change of GBTC cases from 1990 to 2019 by SDI and age in females

Supplementary Table S10. The percentage change of GBTC cases from 1990 to 2019 by SDI and age in males

Supplementary Figure S1. The trend of the number of incident cases (A), prevalent cases (B), deaths (C), and DALYs (D) due to gallbladder and biliary tract cancer by age from 1990 to 2019.

Supplementary Figure S2. The correlation between ASR and HDI in 2019. (A) The age-standardized incidence positively associated with the HDI; (B) The age-standardized prevalence positively associated with the HDI; (C) The age-standardized mortality positively associated with the HDI; (D) The age-standardized rate of DALYs positively associated with the HDI when HDI score was limited to below 0.82, whereas a significant negative association was found for a HDI

score above 0.82. The circles represent countries that were available on HDI data. The size of each circle is proportional to the number of incident cases, prevalent cases, deaths, and DALYs, respectively. The ρ indices and p values were derived from Pearson correlation analysis. ASR, age-standardized rate; GBTC, Gallbladder and biliary tract cancer

Supplementary Figure S3. The risk factors of gallbladder and biliary tract cancer.

Supplementary Table S1. The incident cases and age-standardized incidence of GBTC in 1990 and 2019, and its temporal trends from 1990 to 2019

Characteristics	1990		2019		1990~2019
	Incident case No. ×10^3 (95% UI)	ASR per 100,000 No. (95% UI)	Incident case No. ×10^3 (95% UI)	ASR per 100,000 No. (95% UI)	EAPC No. (95% CI)
**Overall**	107.8 (96.9-119.9)	2.9 (2.6-3.2)	199.2 (166.8-219.6)	2.5 (2.1-2.7)	−0.48 (−0.55-−0.4)
**Sex**					
Male	40.4 (36.4-45.4)	2.5(2.2-2.7)	86.4 (69.4-95.9)	2.4 (1.9-2.7)	0.04 (−0.06-0.15)
Female	67.4 (57.8-78.7)	3.3 (2.8-3.8)	112.8 (9.2-13.0)	2.6 (2.1-3.0)	−0.82 (−0.87-−0.76)
**Socio-demographic index**					
High	44.9 (36.4-47.0)	4.2 (3.4-4.4)	63.8 (52.1-72.3)	3.2 (2.6-3.6)	−1.09 (−1.14-−1.03)
High -middle	29.9 (26.7-32.4)	2.9 (2.6-3.2)	50.8 (37.7-57.5)	2.5 (1.9-2.8)	−0.48 (−0.6-−0.36)
Middle	18.3 (16.4-23.9)	1.9 (1.7-2.5)	45.8 (39.6-53.4)	1.9 (1.7-2.2)	0.23 (0.11-0.34)
Low-middle	10.9 (9.1-15.6)	1.9 (1.6-2.7)	29.6 (26.0 -33.9)	2.2 (2-2.6)	0.58 (0.51-0.66)
Low	3.7 (2.9-4.8)	1.6 (1.3-2.2)	9.1 (7.7-10.5)	1.9 (1.6-2.1)	0.54 (0.45-0.64)
**Region**					
High-income Asia Pacific	18.1 (14.8-19.1)	9.3 (7.6-9.9)	32.8 (24.2-38.6)	6.4 (4.9-7.4)	−1.45 (-1.49-−1.41)
Central Asia	0.5 (0.4-0.6)	1.1 (0.9-1.3)	0.7 (0.6-0.9)	1.1 (0.9-1.3)	−0.09 (−0.2-0.02)
East Asia	13.3 (11.1-21.1)	1.6 (1.4-2.6)	40.5 (29.2-48.5)	2 (1.5-2.4)	1.44 (1.02-1.86)
South Asia	10.7 (8.9-15.2)	2 (1.7-2.9)	35.7 (28.0-40.9)	2.6 (2.1-3)	0.96 (0.81-1.11)
Southeast Asia	4.5 (3.6-5.2)	1.9 (1.5-2.2)	11.4 (9.1-13.9)	2 (1.6-2.4)	0.18 (0.08-0.28)
Australasia	0.6 (0.5-0.7)	2.4 (2.2-2.8)	0.9 (0.7-1.1)	1.8 (1.4-2.1)	−1.19 (−1.26-−1.12)
Caribbean	0.8 (0.5-0.9)	3.2 (2-3.5)	0.9 (0.7-1.1)	1.7 (1.4-2.1)	−2.41 (−2.92-−1.9)
Central Europe	6.7 (5.7-7.2)	4.6 (3.9-4.9)	6.4 (5.4-7.4)	2.9 (2.5-3.4)	−1.71 (−1.76-−1.66)
Eastern Europe	4.7 (3.9-5.1)	1.7 (1.4-1.9)	5.1 (4.3-5.9)	1.5 (1.3-1.7)	−0.77 (−0.96-−0.57)
Western Europe	22.4 (17.7-23.7)	3.8 (3-4)	23.5 (19.3-27.4)	2.4 (2-2.8)	−1.63 (−1.71-−1.56)
Andean Latin America	1.1 (0.9-1.2)	5.5 (4.6-6.2)	2.5 (2.0-3.2)	4.6 (3.6-5.8)	−0.68 (−0.8-−0.56)
Central Latin America	3.5 (2.7-3.7)	4.4 (3.3-4.7)	5.9 (4.9-7.8)	2.5 (2.1-3.3)	−2.27 (−2.42-−2.12)
Southern Latin America	4.4 (3.5-4.7)	9.6 (7.6-10.3)	5.3 (4.1-7.1)	6.3 (4.9-8.4)	−1.62 (−1.69-−1.55)

Tropical Latin America	3.1 (2.6-3.4)	3.6 (3-3.9)	5.7 (4.9-6.6)	2.4 (2.1-2.8)	−1.41 (−1.48-−1.33)
North Africa and Middle East	3.1 (2.5-3.8)	1.9 (1.5-2.4)	6.9 (6.0-8.3)	1.7 (1.5-2.1)	−0.39 (−0.43-−0.36)
High-income North America	7.9 (6.6-8.4)	2.2 (1.8-2.3)	10.7 (9.1-13.1)	1.7 (1.4-2.1)	−0.97 (−1.02-−0.93)
Oceania	0.03 (0.02-0.04)	1.1 (0.9-1.3)	0.07 (0.05-0.09)	1 (0.8-1.3)	−0.28 (−0.31-−0.25)
Central Sub-Saharan Africa	0.2 (0.2-0.3)	1.2 (0.9-1.5)	0.5 (0.4-0.7)	1 (0.8-1.4)	−0.48 (−0.53-−0.42)
Eastern Sub-Saharan Africa	1.0 (0.8-1.2)	1.3 (1.1-1.6)	1.8 (1.5-2.2)	1.2 (1-1.5)	−0.33 (−0.38-−0.29)
Southern Sub-Saharan Africa	0.2 (0.2-0.3)	0.9 (0.8-1.1)	0.5 (0.4-0.6)	0.9 (0.8-1)	−0.04 (−0.26-0.18)
Western Sub-Saharan Africa	0.9 (0.7-1.2)	1.1 (0.9-1.4)	1.6 (1.3-2.4)	1 (0.8-1.4)	−0.34 (−0.42-−0.26)

Supplementary Table S2. The prevalent cases and age-standardized prevalence of GBTC in 1990 and 2019, and its temporal trends from 1990 to 2019

Characteristics	1990		2019		1990~2019
	Prevalent case No. ×10^3 (95% UI)	ASR per 100,000 No. (95% UI)	Prevalent case No. ×10^3 (95% UI)	ASR per 100,000 No. (95% UI)	EAPC No. (95% CI)
**Overall**	133.7 (119.8-147.0)	3.5 (3.1-3.8)	256.3 (215.7-282.0)	3.2 (2.7-3.5)	−0.27 (-0.35-−0.19)
**Sex**					
Male	52.17 (46.68-57.43)	3.05 (2.69-3.30)	116.41 (94.57-129.14)	3.16 (2.55-3.50)	0.28 (0.16-0.40)
Female	81.56 (69.20-93.44)	3.88 (3.28-4.45)	139.93 (113.47-160.86)	3.20 (2.62-3.68)	−0.66 (−0.72-−0.60)
**Socio-demographic index**					
High	62.0 (50.5-65.0)	5.9 (4.8-6.2)	94.6 (79. 1-107.4)	4.8 (4.1-5.5)	−0.67 (−0.74-−0.6)
High -middle	35.5 (31.3-38.1)	3.4 (3-3.6)	64.7 (47.4-73.5)	3.2 (2.3-3.6)	−0.13 (−0.25-0)
Middle	20.4 (18.3-26.4)	2 (1.8-2.6)	54.2 (46.7-62.7)	2.2 (1.9-2.6)	0.53 (0.41-0.64)
Low-middle	11.8 (9.9-17.0)	2 (1.7-2.8)	32.8 (28.7-37.6)	2.4 (2.1-2.7)	0.74 (0.67-0.8)
Low	3.9 (3.1 -5.2)	1.6 (1.3-2.2)	9.9 (8.3-11.4)	1.9 (1.6-2.2)	0.67 (0.58-0.76)
**Region**					
High-income Asia Pacific	23.1 (18.8-24.5)	11.7 (9.5-12.4)	43.6 (32.6-52.0)	8.9 (7-10.5)	−0.97 (−1.03-−0.9)
Central Asia	0.6 (0.5-0.6)	1.2 (1-1.4)	0.8 (0.7-1.0)	1.1 (0.9-1.3)	−0.14 (−0.24-−0.03)
East Asia	14.8 (12.3-23.2)	1.7 (1.4-2.7)	49.8 (35.1-59.8)	2.4 (1.7-2.9)	1.96 (1.53-2.39)
South Asia	11.7 (9.6-16.6)	2.1 (1.7-2.9)	39.5 (30.9-45.4)	2.8 (2.2-3.2)	1.15 (1.01-1.28)
Southeast Asia	5.1 (4.1-5.9)	2 (1.6-2.3)	14.0 (10.9-17.2)	2.3 (1.8-2.9)	0.56 (0.46-0.65)
Australasia	0.8 (0.7-0.9)	3.4 (3-3.9)	1.4 (1.1-1.7)	2.7 (2.2-3.3)	−0.73 (−0.82-−0.65)
Caribbean	0.9 (0.6-1.0)	3.5 (2.2-3.9)	1.0 (0.8-1.2)	1.9 (1.6-2.4)	−2.31 (−2.82-−1.79)
Central Europe	7.4 (6.3-7.9)	5 (4.3-5.3)	7.3 (6.2-8.5)	3.4 (2.9-4)	−1.44 (−1.48-−1.39)
Eastern Europe	5.6 (4.6-6.0)	2 (1.7-2.2)	6.6 (5.6-7.6)	1.9 (1.6-2.2)	−0.31 (−0.45-−0.17)
Western Europe	31.0 (24.6-32.9)	5.3 (4.2-5.6)	36.4 (30,1-42.4)	4 (3.3-4.6)	−1.07 (−1.13-−1.01)
Andean Latin America	1.2 (1.0-1.4)	5.9 (4.9-6.6)	3.0 (2.3-3.8)	5.4 (4.2-6.9)	−0.36 (−0.47-−0.24)
Central Latin America	4.0 (3.0-4.2)	4.8 (3.6-5.1)	6.9 (5.7-9.0)	2.9 (2.4-3.8)	−2.08 (−2.23-−1.92)
Southern Latin America	5.0 (4.0-5.3)	10.8 (8.6-11.6)	6.4 (5.0-8.5)	7.8 (6-10.3)	−1.31 (−1.38-−1.24)

Tropical Latin America	3.5 (2.9-3.8)	3.9 (3.2-4.2)	6.6 (5.7-7.6)	2.7 (2.4-3.1)	−1.21 (−1.28-−1.13)
North Africa and Middle East	3.5 (2.8-4.3)	2 (1.6-2.5)	8.2 (7.1-10.0)	1.9 (1.7-2.4)	−0.17 (−0.21-−0.13)
High-income North America	13.3 (11.1-14.0)	3.7 (3.1-3.9)	20.0 (17.1-24.2)	3.2 (2.8-3.9)	−0.55 (−0.61-−0.48)
Oceania	0.03 (0.03-0.04)	1.1 (0.9-1.4)	0.08 (0.06-0.10)	1.1 (0.8-1.4)	−0.17 (−0.2-−0.14)
Central Sub-Saharan Africa	0.3 (0.2-0.3)	1.2 (0.9-1.5)	0.5 (0.4-0.7)	1 (0.8-1.4)	−0.40 (−0.47-−0.33)
Eastern Sub-Saharan Africa	1.0 (0.8-1.2)	1.3 (1.1-1.6)	2.0 (1.6-2.4)	1.2 (1-1.5)	−0.23 (−0.29-−0.16)
Southern Sub-Saharan Africa	0.3 (0.2-0.3)	1 (0.8-1.2)	0.5 (0.5-0.6)	1 (0.8-1.1)	−0.03 (−0.2-0.13)
Western Sub-Saharan Africa	0.9 (0.8-1.2)	1.1 (0.9-1.4)	1.7 (1.4-2.6)	1 (0.8-1.4)	−0.27 (−0.37-−0.17)

Supplementary Table S3. The number of death and age-standardized mortality rate of GBTC in 1990 and 2019, and its temporal trends from 1990 to 2019

Characteristics	1990		2019		1990~2019
	Deaths No. ×10^3 (95% UI)	ASR per 100,000 No. (95% UI)	Deaths No. ×10^3 (95% UI)	ASR per 100,000 No. (95% UI)	EAPC No. (95% CI)
**Overall**	94.86 (85.14-107.24)	2.6 (2.3-2.9)	172.44 (144.90-188.62)	2.2 (1.8-2.4)	−0.58 (−0.65-−0.51)
**Sex**					
Male	34.83 (30.88-40.06)	2.2 (1.9-2.5)	73.00 (59.48-80.40)	2.1 (1.7-2.3)	−0.07 (−0.17-0.04)
Female	60.02 (51.25-70.18)	2.9 (2.5-3.4)	99.45 (81.73-114.00)	2.3 (1.9-2.6)	−0.86 (−0.97-−0.75)
**Socio-demographic index**					
High	35.59 (28.73-37.29)	3.4 (2.7-3.5)	48.41 (38.29-53.44)	2.3 (1.9-2.5)	−1.38 (−1.44-−1.33)
High -middle	27.26 (24.42-29.59)	2.7 (2.4-3.0)	44.04 (33.51-49.35)	2.2 (1.7-2.4)	−0.73 (−0.86-−0.6)
Middle	17.67 (15.79-23.07)	1.9 (1.7-2.5)	42.30 (36.87-49.24)	1.8 (1.6-2.1)	0 (−0.11-0.12)
Low-middle	10.65 (8.95-15.39)	2 (1.7-2.8)	28.68 (25.26-33.09)	2.2 (2-2.6)	0.49 (0.4-0.58)
Low	3.64 (2.92-4.83)	1.7 (1.4-2.3)	8.95 (7.45-10.26)	1.9 (1.6-2.2)	0.49 (0.39-0.59)
**Region**					
High-income Asia Pacific	15.45 (12.65-16.28)	8 (6.6-8.5)	27.13 (19.58-30.77)	5.1 (3.8-5.7)	−1.79 (−1.84-−1.74)
Central Asia	0.48 (0.41-0.57)	1.1 (0.9-1.3)	0.71 (0.59-0.83)	1.1 (0.9-1.2)	−0.03 (−0.15-0.09)
East Asia	12.80 (10.62-20.45)	1.6 (1.4-2.6)	36.03 (26.79-42.82)	1.8 (1.4-2.2)	1 (0.59-1.41)
South Asia	10.47 (8.60-14.96)	2.1 (1.7-3)	34.54 (26.64-39.59)	2.6 (2-3)	0.84 (0.68-1.01)
Southeast Asia	4.34 (3.49-5.03)	1.9 (1.5-2.2)	10.25 (8.30-12.44)	1.9 (1.5-2.3)	−0.09 (−0.19-0.02)
Australasia	0.44 (0.39-0.51)	1.9 (1.7-2.2)	0.65 (0.54-0.74)	1.3 (1-1.4)	−1.5 (−1.57-−1.44)
Caribbean	0.77 (0.49-0.86)	3 (1.9-3.4)	0.81 (0.68-1.01)	1.6 (1.3-2)	−2.46 (−2.96-−1.95)
Central Europe	6.48 (5.52-6.94)	4.5 (3.8-4.8)	6.10 (5.24-7.13)	2.8 (2.4-3.2)	−1.83 (−1.89-−1.78)
Eastern Europe	4.15 (3.49-4.59)	1.5 (1.3-1.7)	4.24 (3.53-4.90)	1.2 (1-1.4)	−1.12 (−1.34-−0.89)
Western Europe	17.60 (13.81-18.49)	3 (2.3-3.1)	16.99 (14.15-19.08)	1.7 (1.4-1.9)	−2.04 (−2.13-−1.95)
Andean Latin America	1.06 (0.89-1.20)	5.4 (4.6-6.2)	2.32 (1.83-2.93)	4.2 (3.3-5.4)	−0.92 (−1.05-−0.8)
Central Latin America	3.37 (2.56-3.57)	4.3 (3.3-4.6)	5.50 (4.65-7.36)	2.4 (2-3.2)	−2.39 (−2.53-−2.24)
Southern Latin America	4.08 (3.24-4.35)	9.1 (7.2-9.7)	4.77 (4.30-6.22)	5.7 (5.1-7.4)	−1.84 (−1.91-−1.77)

Tropical Latin America	2.96 (2.46-3.21)	3.5 (2.9-3.8)	5.40 (4.69-6.30)	2.3 (2-2.7)	−1.52 (−1.6-−1.45)
North Africa and Middle East	2.96 (2.39-3.70)	1.9 (1.5-2.4)	6.27 (5.47-7.71)	1.6 (1.4-2)	−0.56 (−0.6-−0.53)
High-income North America	5.11 (4.25-5.44)	1.4 (1.2-1.5)	6.25 (5.60-7.57)	1 (0.9-1.2)	−1.37 (−1.43-−1.31)
Oceania	0.03 (0.02-0.04)	1.1 (0.9-1.4)	0.06 (0.05-0.08)	1 (0.8-1.3)	−0.36 (−0.4-−0.33)
Central Sub-Saharan Africa	0.25 (0.19-0.31)	1.2 (1-1.5)	0.49 (0.36-0.67)	1.1 (0.8-1.4)	−0.5 (−0.54-−0.45)
Eastern Sub-Saharan Africa	0.95 (0.75-1.18)	1.4 (1.1-1.7)	1.84 (1.51-2.22)	1.3 (1-1.5)	−0.23 (−0.27-−0.19)
Southern Sub-Saharan Africa	0.24 (0.21-0.29)	1 (0.8-1.1)	0.48 (0.40-0.55)	0.9 (0.8-1.1)	−0.01 (−0.24-0.22)
Western Sub-Saharan Africa	0.90 (0.74-1.18)	1.2 (1-1.5)	1.64 (1.35-2.41)	1 (0.8-1.5)	−0.36 (−0.43-−0.28)

Supplementary Table S4. The number and age-standardized rate of DALYs due to GBTC in 1990 and 2019, and its temporal trends from 1990 to 2019

Characteristics	1990		2019		1990~2019
	DALYs No. ×10[3] (95% UI)	ASR per 100,000 No. (95% UI)	DALYs No. ×10[3] (95% UI)	ASR per 100,000 No. (95% UI)	EAPC No. (95% CI)
**Overall**	2153.57 (1933.11-2455.89)	54.1 (48.7-61.3)	3621.47 (3102.42-3969.07)	44 (37.6-48.2)	−0.67 (−0.74-−0.61)
**Sex**					
**Male**	834.09 (744.53-961.35)	45.26 (40.17-52.26)	1579.00 (1301.03-1756.59)	41.02 (33.68-45.29)	−0.20 (−0.31-−0.10)
**Female**	1319.48 (1124.24-1538.82)	61.78 (52.66-72.27)	2042.47 (1688.64-2354.41)	46.83 (38.70-53.98)	−0.94 (−1.05-−0.83)
**Socio-demographic index**					
High	711.08 (583.11-740.63)	68.7 (56.5-71.6)	787.80 (665.13-866.23)	42.1 (36.3-46.7)	−1.85 (−1.91-−1.8)
High -middle	619.22 (550.01-662.65)	57.5 (51.2-61.8)	920.02 (690.25-1035.01)	45.1 (33.9-50.8)	−0.79 (-0.92-−0.66)
Middle	447.64 (400.26-581.27)	42 (37.6-54.6)	976.54 (853.01-1140.02)	38.4 (33.6-44.7)	−0.1 (−0.21-0.01)
Low-middle	277.85 (231.75-404.84)	44.4 (37.3-64.4)	706.87 (613.06-809.04)	50.1 (43.6-57.3)	0.52 (0.43-0.6)
Low	96.63 (75.79-128.77)	38.8 (30.8-51.4)	228.77 (187.59-263.49)	42.3 (35.1-48.6)	0.41 (0.3-0.52)
**Region**					
High-income Asia Pacific	318.74 (260.44-334.87)	157.6 (129.1-165.7)	398.51 (311.25-441.61)	88.4 (74.2-98.3)	−2.24 (-2.3-−2.17)
Central Asia	11.69 (10.04-13.50)	24.6 (21-28.6)	17.85 (14.54-20.73)	23.4 (19.3-27.2)	−0.23 (-0.35-−0.1)
East Asia	327.93 (271.27-517.93)	35.9 (29.9-56.9)	798.90 (604.40-955.61)	38.1 (28.7-45.5)	0.82 (0.42-1.22)
South Asia	279.90 (229.32-400.38)	46.9 (38.5-67)	865.13 (658.25-992.59)	59.3 (45.4-68)	0.92 (0.77-1.07)
Southeast Asia	110.51 (88.42-128.61)	41.6 (33.4-48.4)	238.94 (192.00-289.27)	38.7 (31.2-46.8)	−0.31 (−0.4-−0.21)
Australasia	9.06 (8.02-10.43)	38.7 (34.2-44.5)	11.63 (9.83-13.18)	24.3 (20.7-27.5)	−1.68 (−1.74-−1.62)
Caribbean	17.94 (11.35-20.22)	67.9 (43.2-76.5)	18.73 (15.35-23.43)	36.2 (29.7-45.2)	−2.35 (−2.86-−1.84)
Central Europe	140.43 (119.44-149.41)	94.3 (80.4-100.5)	118.88 (102.19-139.38)	56.7 (48.7-66.1)	−1.88 (−1.92-−1.84)
Eastern Europe	94.92 (78.31-103.25)	33.5 (27.7-36.6)	91.66 (77.01-105.84)	27.1 (22.8-31.3)	−1.17 (−1.43-−0.91)
Western Europe	334.62 (263.40-349.47)	58.5 (46.1-61.1)	282.85 (240.41-324.01)	32.3 (27.9-37.5)	−2.2 (−2.28-−2.12)
Andean Latin America	25.75 (21.57-29.40)	122 (102.6-138.9)	50.54 (39.37-64.54)	89.8 (69.9-114.1)	−1.14 (−1.27-−1.01)
Central Latin America	81.95 (62.22-86.61)	95.2 (72.4-100.7)	123.23 (104.06-164.75)	51.6 (43.6-69.1)	−2.45 (−2.61-−2.29)
Southern Latin America	91.43 (72.47-97.21)	197 (156.1-209.7)	99.48 (90.62-125.64)	121.3 (110.8-152.7)	−1.86 (−1.93-−1.79)

Tropical Latin America	72.54 (61.32-79.79)	77 (64.5-84.1)	121.49 (106.80-139.52)	49.4 (43.4-56.9)	−1.53 (-1.6-−1.46)
North Africa and Middle East	75.86 (60.84-91.66)	42.3 (34-52.2)	152.33 (129.01-180.64)	33.9 (29.4-40.9)	−0.78 (−0.81-−0.75)
High-income North America	100.30 (85.53-107.23)	29 (24.7-31)	119.33 (109.84-142.83)	19.7 (18.2-23.6)	−1.4 (−1.46-−1.33)
Oceania	0.79 (0.61-0.99)	25.1 (19.5-30.7)	1.71 (1.24-2.27)	22.7 (16.8-29.3)	−0.33 (−0.36-−0.3)
Central Sub-Saharan Africa	6.60 (4.81-8.60)	27.5 (20.8-35)	13.08 (9.60-17.92)	23.3 (17.2-31.5)	−0.55 (−0.59-−0.51)
Eastern Sub-Saharan Africa	25.42 (19.59-31.79)	31.8 (24.9-39.5)	46.65 (37.85-57.00)	27.5 (22.7-33.5)	−0.52 (−0.56-−0.48)
Southern Sub-Saharan Africa	5.96 (5.22-6.89)	20.8 (18-24.4)	11.41 (9.69-13.19)	19.7 (16.7-22.8)	−0.02 (−0.24-0.19)
Western Sub-Saharan Africa	21.26 (17.31-27.62)	24.2 (19.8-31.6)	39.17 (31.74-56.66)	20.8 (17.1-30.6)	−0.42 (−0.5-−0.35)

Supplementary Table S5. The number and age-standardized rate of YLDs due to GBTC in 1990 and 2019, and its temporal trends from 1990 to 2019

Characteristics	1990		2019		1990~2019
	YLDs No. ×10^3 (95% UI)	ASR per 100,000 No. (95% UI)	YLDs No. ×10^3 (95% UI)	ASR per 100,000 No. (95% UI)	EAPC No. (95% CI)
**Overall**	25.85 (18.46-33.87)	0.7 (0.5-0.9)	48.44 (33.77-64.79)	0.6 (0.4-0.8)	−0.39 (−0.47-−0.32)
**Sex**					
**Male**	9.93 (7.05-13.06)	0.59 (0.42-0.78)	21.40 (14.45-28.55)	0.59 (0.42-0.78)	0.10 (−0.01-0.21)
**Female**	15.91 (10.98-21.24)	0.76 (0.53-1.02)	27.04 (18.54-36.34)	0.62 (0.42-0.83)	−0.73 (−0.79-−0.67)
**Socio-demographic index**					
High	10.83 (7.61-14.15)	1 (0.7-1.3)	15.54 (10.66-20.83)	0.8 (0.5-1)	−0.98 (−1.04-−0.93)
High -middle	7.13 (5.04-9.38)	0.7 (0.5-0.9)	12.39 (8.03-16.78)	0.6 (0.4-0.8)	−0.35 (−0.48-−0.23)
Middle	4.38 (3.06-6.05)	0.4 (0.3-0.6)	11.16 (7.61-14.95)	0.5 (0.3-0.6)	0.33 (0.21-0.45)
Low-middle	2.61 (1.74-3.88)	0.5 (0.3-0.7)	7.13 (4.86-955)	0.5 (0.4-0.7)	0.63 (0.56-0.7)
Low	0.89 (0.59-1.27)	0.4 (0.3-0.6)	2.19 (1.49-2.96)	0.4 (0.3-0.6)	0.55 (0.46-0.65)
**Region**					
High-income Asia Pacific	4.14 (2.90-5.38)	2.1 (1.5-2.7)	7.38 (4.76-10.02)	1.5 (1-2)	−1.3 (−1.35-−1.25)
Central Asia	0.12 (0.08-0.17)	0.3 (0.2-0.4)	0.18 (0.12-0.25)	0.3 (0.2-0.4)	−0.1 (−0.2-0.01)
East Asia	3.21 (2.15-5.13)	0.4 (0.3-0.6)	9.94 (6.23-13.78)	0.5 (0.3-0.7)	1.49 (1.08-1.9)
South Asia	2.57 (1.73-3.78)	0.5 (0.3-0.7)	8.58 (5.68-11.70)	0.6 (0.4-0.8)	1 (0.86-1.13)
Southeast Asia	1.08 (0.72-1.46)	0.4 (0.3-0.6)	2.77 (1.80-3.90)	0.5 (0.3-0.7)	0.26 (0.17-0.35)
Australasia	0.15 (0.10-0.19)	0.6 (0.4-0.8)	0.23 (0.15-0.33)	0.5 (0.3-0.7)	−1.06 (−1.12-−0.99)
Caribbean	0.20 (0.11-0.27)	0.8 (0.4-1)	0.21 (0.14-0.30)	0.4 (0.3-0.6)	−2.34 (−2.84-−1.84)
Central Europe	1.54 (1.07-2.03)	1 (0.7-1.4)	1.52 (1.01-2.08)	0.7 (0.5-0.9)	−1.53 (−1.57-−1.49)
Eastern Europe	1.16 (0.79-1.58)	0.4 (0.3-0.6)	1.29 (0.87-1.80)	0.4 (0.3-0.5)	−0.65 (-0.84-−0.47)
Western Europe	5.46 (3.77-7.18)	0.9 (0.6-1.2)	5.97 (4.04-8.10)	0.6 (0.4-0.9)	−1.41 (−1.47-−1.35)
Andean Latin America	0.25 (0.17-0.34)	1.3 (0.9-1.7)	0.59 (0.38-0.84)	1.1 (0.7-1.5)	−0.6 (−0.71-−0.48)
Central Latin America	0.83 (0.55-1.10)	1 (0.7-1.4)	1.43 (0.96-2.02)	0.6 (0.4-0.9)	−2.08 (−2.22-−1.94)
Southern Latin America	0.98 (0.67-1.30)	2.1 (1.5-2.8)	1.22 (0.78-1.81)	1.5 (0.9-2.2)	−1.46 (−1.52-−1.39)

Tropical Latin America	0.74 (0.51-0.98)	0.8 (0.6-1.1)	1.39 (0.96-1.87)	0.6 (0.4-0.8)	−1.27 (−1.34-−1.2)
North Africa and Middle East	0.75 (0.50-1.01)	0.5 (0.3-0.6)	1.68 (1.16-2.28)	0.4 (0.3-0.6)	−0.36 (−0.39-−0.32)
High-income North America	2.12 (1.48-2.83)	0.6 (0.4-0.8)	2.96 (1.99-4.08)	0.5 (0.3-0.6)	−0.84 (−0.89-−0.79)
Oceania	0.007 (0.005-0.01)	0.3 (0.2-0.4)	0.02 (0.01-0.024)	0.2 (0.2-0.3)	−0.26 (−0.29-−0.23)
Central Sub-Saharan Africa	0.06 (0.04-0.09)	0.3 (0.2-0.4)	0.12 (0.08-0.18)	0.2 (0.2-0.4)	−0.47 (−0.52-−0.41)
Eastern Sub-Saharan Africa	0.23 (0.15-0.32)	0.3 (0.2-0.4)	0.44 (0.30-0.62)	0.3 (0.2-0.4)	−0.31 (−0.36-−0.26)
Southern Sub-Saharan Africa	0.06 (0.04-0.08)	0.2 (0.2-0.3)	0.12 (0.08-0.16)	0.2 (0.1-0.3)	−0.04 (−0.25-0.17)
Western Sub-Saharan Africa	0.21 (0.14-0.29)	0.3 (0.2-0.4)	0.39 (0.26-0.57)	0.2 (0.2-0.3)	−0.33 (−0.42—0.25)

Supplementary Table S6. The number and age-standardized rate of YLLs due to GBTC in 1990 and 2019, and its temporal trends from 1990 to 2019

Characteristics	1990		2019		1990~2019
	YLLs No. ×10^3 (95% UI)	ASR per 100,000 No. (95% UI)	YLLs No. ×10^3 (95% UI)	ASR per 100,000 No. (95% UI)	EAPC No. (95% CI)
**Overall**	2127.72 (1914.12-2429.00)	53.4 (48.1-60.8)	3573.04 (3063.59-3916.20)	43.4 (37.1-47.6)	−0.67 (−0.74-−0.61)
**Sex**					
**Male**	824.16 (735.69-951.30)	44.64 (39.62-51.39)	1557.61 (1283.43-1728.06)	40.43 (33.20-44.64)	−0.21 (−0.31-−0.11)
**Female**	1303.56 (1111.15-1524.51)	61.02 (52.04-71.23)	2015.43 (1665.18-2328.25)	46.21 (38.17-53.35)	−0.97 (−1.04-−0.91)
**Socio-demographic index**					
High	700.26 (574.23-728.25)	67.7 (55.7-70.4)	772.26 (654.01-848.08)	41.3 (35.7-45.7)	−1.87 (−1.93-−1.81)
High -middle	612.09 (543.57-655.38)	56.8 (50.6-61.1)	907.62 (682.25-1021.01)	44.5 (33.5-50.1)	−0.8 (−0.93-−0.67)
Middle	443.26 (396.41-574.89)	41.6 (37.2-54)	965.38 (843.57-1128.25)	38 (33.2-44.2)	−0.1 (−0.22-0.01)
Low-middle	275.24 (229.52-401.29)	44 (36.9-63.7)	699.74 (607.50-802.34)	49.6 (43.2-56.7)	0.51 (0.43-0.59)
Low	95.75 (75.16-127.29)	38.4 (30.5-51)	226.58 (185.66-261.45)	41.9 (34.7-48)	0.41 (0.3-0.52)
**Region**					
High-income Asia Pacific	314.59 (256.93-330.63)	155.5 (127.6-163.4)	391.13 (306.53-433.54)	86.9 (72.9-96.9)	−2.25 (−2.31-−2.19)
Central Asia	11.57 (9.95-13.37)	24.3 (20.8-28.3)	17.67 (14.40-20.52)	23.2 (19.1-26.8)	−0.23 (−0.35-−0.1)
East Asia	324.72 (268.48-512.77)	35.5 (29.5-56.2)	788.95 (594.54-942.22)	37.6 (28.2-44.9)	0.81 (0.41-1.21)
South Asia	277.32 (227.14-396.71)	46.5 (38.2-66.3)	856.55 (650.65-985.36)	58.7 (44.8-67.4)	0.92 (0.77-1.07)
Southeast Asia	109.43 (87.46-127.30)	41.2 (33-47.8)	236.17 (189.90-285.64)	38.2 (30.8-46.4)	−0.31 (−0.41-−0.22)
Australasia	8.91 (7.89-10.26)	38.1 (33.6-43.8)	11.40 (9.63-12.91)	23.8 (20.3-26.9)	−1.69 (−1.75-−1.63)
Caribbean	17.74 (11.25-20.00)	67.2 (42.8-75.7)	18.52 (15.23-23.15)	35.8 (29.5-44.7)	−2.35 (−2.86-−1.84)
Central Europe	138.89 (118.22-148.00)	93.3 (79.6-99.4)	117.36 (100.84-137.47)	56 (48-65.4)	−1.88 (−1.93-−1.84)
Eastern Europe	93.76 (77.48-102.18)	33.1 (27.4-36.2)	90.37 (75.94-104.40)	26.7 (22.5-30.9)	−1.18 (−1.44-−0.92)
Western Europe	329.16 (259.24-343.62)	57.6 (45.4-60.1)	276.88 (235.44-317.21)	31.7 (27.4-36.7)	−2.21 (−2.3-−2.13)
Andean Latin America	25.50 (21.39-29.13)	120.8 (101.6-137.5)	49.95 (38.89-63.66)	88.7 (69.2-112.5)	−1.15 (−1.28-−1.02)
Central Latin America	81.12 (61.52-85.78)	94.2 (71.7-99.7)	121.80 (102.84-162.76)	51 (43-68.2)	−2.45 (−2.62-−2.29)
Southern Latin America	90.46 (71.77-96.12)	194.9 (154.3-207.4)	98.26 (89.52-124.07)	119.9 (109.5-150.8)	−1.86 (−1.94-−1.79)

Tropical Latin America	71.80 (60.72-78.98)	76.2 (63.8-83.2)	120.10 (105.75-137.99)	48.8 (43-56.3)	−1.53 (−1.6-−1.46)
North Africa and Middle East	75.11 (60.21-90.75)	41.8 (33.6-51.7)	150.64 (127.73-178.66)	33.5 (29-40.4)	−0.78 (−0.81-−0.76)
High-income North America	98.18 (83.55-104.83)	28.4 (24.2-30.3)	116.36 (106.95-139.73)	19.2 (17.7-23.1)	−1.41 (−1.47-−1.35)
Oceania	0.78 (0.60-0.98)	24.8 (19.3-30.4)	1.70 (1.23-2.25)	22.4 (16.6-29)	−0.33 (−0.36-−0.3)
Central Sub-Saharan Africa	6.54 (4.77-8.52)	27.2 (20.6-34.6)	12.96 (9.51-17.78)	23.1 (17-31.2)	−0.55 (−0.59-−0.51)
Eastern Sub-Saharan Africa	25.19 (19.40-31.54)	31.4 (24.7-39.1)	46.21 (37.48-56.50)	27.3 (22.4-33.1)	−0.52 (−0.56-−0.48)
Southern Sub-Saharan Africa	5.90 (5.17-6.82)	20.6 (17.8-24.1)	11.30 (9.61-13.04)	19.5 (16.6-22.5)	−0.02 (−0.24-0.19)
Western Sub-Saharan Africa	21.05 (17.12-27.36)	23.9 (19.6-31.3)	38.78 (31.41-56.09)	20.6 (16.9-30.3)	−0.43 (−0.5-−0.35)

Supplementary Table S7. The change of GBTC cases between 1990 and 2019 at national level, both sexes

Location	Incidence		Prevalence		Deaths		DALYs	
	Percentage change in absolute number (%)	EAPC No. (95% CI)	Percentage change in absolute number (%)	EAPC No. (95% CI)	Percentage change in absolute number (%)	EAPC No. (95% CI)	Percentage change in absolute number (%)	EAPC No. (95% CI)
**Afghanistan**	62.17	-0.23 (-0.3--0.16)	71.78	-0.17 (-0.24--0.1)	58.17	-0.23 (-0.3--0.15)	72.81	-0.37 (-0.48--0.26)
**Albania**	90.80	-0.03 (-0.4-0.35)	91.31	0.11 (-0.29-0.51)	90.37	-0.11 (-0.47-0.25)	66.38	-0.13 (-0.5-0.25)
**Algeria**	157.61	-0.26 (-0.34--0.18)	167.49	-0.13 (-0.19--0.07)	151.81	-0.34 (-0.44--0.24)	131.76	-0.57 (-0.66--0.48)
**American Samoa**	42.85	-1.73 (-1.91--1.55)	45.36	-1.53 (-1.74--1.32)	40.56	-1.9 (-2.06--1.74)	32.55	-1.81 (-1.99--1.63)
**Andorra**	115.95	-0.93 (-1.02--0.83)	116.13	-0.7 (-0.78--0.62)	121.55	-1.01 (-1.11--0.91)	88.64	-1.07 (-1.16--0.97)
**Angola**	162.33	-0.22 (-0.27--0.17)	171.81	-0.1 (-0.16--0.03)	158.24	-0.24 (-0.29--0.2)	150.67	-0.42 (-0.47--0.38)
**Antigua and Barbuda**	-26.98	-3.42 (-4.02--2.82)	-23.05	-3.37 (-3.99--2.75)	-29.38	-3.43 (-4.02--2.84)	-24.74	-3.64 (-4.24--3.04)
**Argentina**	9.82	-1.68 (-1.82--1.53)	16.08	-1.4 (-1.54--1.27)	5.56	-1.86 (-2--1.72)	3.53	-1.68 (-1.79--1.58)
**Armenia**	112.08	1.16 (0.67-1.64)	105.47	1.21 (0.7-1.72)	118.03	1.17 (0.7-1.63)	90.50	1.06 (0.58-1.53)
**Australia**	51.54	-1.38 (-1.48--1.29)	62.01	-1.01 (-1.12--0.89)	45.03	-1.63 (-1.71--1.55)	25.74	-1.82 (-1.9--1.74)
**Austria**	-29.09	-3.02 (-3.22--2.81)	-19.43	-2.49 (-2.67--2.32)	-35.10	-3.4 (-3.63--3.17)	-40.25	-3.52 (-3.76--3.27)
**Azerbaijan**	98.60	0.59 (0.43-0.75)	96.66	0.42 (0.25-0.58)	99.49	0.74 (0.58-0.9)	95.05	0.19 (0.05-0.34)
**Bahamas**	3.34	-3.24 (-3.74--2.75)	4.92	-3.18 (-3.67--2.69)	2.98	-3.26 (-3.76--2.76)	-0.14	-3.36 (-3.89--2.83)
**Bahrain**	251.66	-1.18 (-1.39--0.97)	301.36	-0.8 (-1.01--0.59)	222.89	-1.35 (-1.58--1.13)	229.95	-1.75 (-1.92--1.58)
**Bangladesh**	186.82	0.24 (0-0.47)	199.02	0.43 (0.23-0.63)	182.22	0.15 (-0.12-0.42)	160.06	0.05 (-0.16-0.25)
**Barbados**	-31.34	-3.36 (-3.89--2.82)	-28.23	-3.26 (-3.8--2.71)	-32.66	-3.39 (-3.92--2.86)	-31.11	-3.5 (-4.04--2.95)
**Belarus**	33.00	0.21 (-0.07-0.49)	35.45	0.33 (0.1-0.56)	30.76	0.12 (-0.19-0.43)	25.22	0.01 (-0.36-0.38)
**Belgium**	-19.01	-2.23 (-2.42--2.03)	-14.94	-1.85 (-2.06--1.64)	-19.31	-2.4 (-2.58--2.23)	-26.16	-2.31 (-2.48--2.13)
**Belize**	28.08	-3.11 (-3.37--2.85)	36.88	-2.98 (-3.25--2.71)	22.24	-3.19 (-3.45--2.94)	38.45	-3.07 (-3.32--2.82)
**Benin**	77.98	-0.91 (-1.01--0.82)	85.35	-0.86 (-0.96--0.75)	74.23	-0.93 (-1.02--0.84)	83.83	-0.97 (-1.06--0.88)
**Bermuda**	-49.06	-5.27 (-5.89--4.65)	-45.88	-4.93 (-5.55--4.31)	-50.88	-5.5 (-6.12--4.88)	-57.91	-5.66 (-6.31--5.01)
**Bhutan**	220.74	1.16 (1.08-1.24)	225.60	1.34 (1.27-1.42)	221.14	1.09 (1-1.18)	170.98	0.76 (0.67-0.85)
**Bolivia**	138.52	-0.63 (-0.76--0.5)	149.99	-0.45 (-0.58--0.32)	131.37	-0.74 (-0.87--0.62)	109.12	-1.04 (-1.17--0.91)
**Bosnia and Herzegovina**	-26.84	-3.38 (-3.65--3.11)	-29.35	-3.35 (-3.63--3.08)	-24.69	-3.38 (-3.64--3.11)	-36.51	-3.64 (-3.94--3.34)

**Botswana**	156.31	0.02 (-0.2-0.24)	172.34	0.15 (-0.04-0.35)	147.06	-0.07 (-0.31-0.17)	155.84	-0.11 (-0.38-0.16)
**Brazil**	84.07	-1.42 (-1.5--1.34)	89.13	-1.22 (-1.29--1.14)	82.09	-1.54 (-1.62--1.46)	66.91	-1.54 (-1.61--1.47)
**Brunei Darussalam**	144.19	-0.28 (-0.45--0.12)	165.26	-0.02 (-0.19-0.14)	131.89	-0.42 (-0.58--0.26)	125.07	-0.67 (-0.87--0.46)
**Bulgaria**	5.56	-0.06 (-0.2-0.08)	6.42	0.13 (-0.03-0.29)	4.48	-0.17 (-0.32--0.03)	-6.76	-0.12 (-0.27-0.03)
**Burkina Faso**	63.94	-0.87 (-1.11--0.64)	69.83	-0.76 (-1.01--0.51)	61.76	-0.9 (-1.13--0.68)	67.61	-0.87 (-1.11--0.62)
**Burundi**	54.53	-0.87 (-0.95--0.8)	64.52	-0.72 (-0.78--0.66)	51.67	-0.85 (-0.93--0.78)	57.45	-1.08 (-1.17--0.98)
**Cabo Verde**	44.83	-1.06 (-1.62--0.49)	54.46	-0.91 (-1.47--0.34)	39.46	-1.16 (-1.71--0.6)	48.56	-1.21 (-1.69--0.72)
**Cambodia**	129.62	-0.5 (-0.69--0.31)	142.03	-0.29 (-0.48--0.09)	123.04	-0.62 (-0.8--0.43)	101.50	-0.93 (-1.12--0.74)
**Cameroon**	104.61	-0.9 (-0.95--0.86)	110.59	-0.82 (-0.89--0.74)	100.56	-0.96 (-1--0.92)	105.09	-0.94 (-0.98--0.9)
**Canada**	39.57	-1.85 (-2--1.7)	44.31	-1.58 (-1.72--1.43)	36.97	-2.02 (-2.17--1.86)	21.24	-2.22 (-2.4--2.04)
**Central African Republic**	55.68	-0.51 (-0.55--0.46)	58.40	-0.48 (-0.51--0.44)	54.79	-0.5 (-0.55--0.46)	59.19	-0.53 (-0.58--0.49)
**Chad**	61.91	-0.51 (-0.55--0.47)	67.83	-0.46 (-0.51--0.41)	58.86	-0.52 (-0.56--0.48)	70.93	-0.48 (-0.52--0.45)
**Chile**	36.17	-2.28 (-2.43--2.12)	46.40	-1.92 (-2.07--1.77)	31.31	-2.53 (-2.69--2.37)	15.57	-2.72 (-2.88--2.56)
**China**	210.66	1.56 (1.12-2)	244.89	2.12 (1.66-2.57)	185.20	1.1 (0.67-1.52)	147.10	0.93 (0.5-1.35)
**Colombia**	78.02	-1.99 (-2.13--1.85)	85.23	-1.72 (-1.86--1.57)	74.88	-2.15 (-2.28--2.02)	51.66	-2.25 (-2.41--2.09)
**Comoros**	124.90	0.05 (-0.03-0.13)	131.10	0.16 (0.07-0.24)	123.68	0.02 (-0.05-0.09)	118.11	-0.09 (-0.2-0.02)
**Republic of Congo**	81.81	-0.95 (-1.07--0.82)	88.98	-0.83 (-0.97--0.69)	79.57	-0.96 (-1.08--0.83)	79.62	-1.14 (-1.27--1.02)
**Cook Islands**	15.10	-2.18 (-2.37--2)	19.27	-1.88 (-2.06--1.7)	11.54	-2.42 (-2.6--2.23)	-2.86	-2.52 (-2.72--2.32)
**Costa Rica**	48.81	-3.1 (-3.36--2.85)	52.43	-2.92 (-3.17--2.68)	47.45	-3.21 (-3.46--2.96)	39.51	-3.21 (-3.46--2.96)
**Côte d'Ivoire**	95.51	-1.19 (-1.26--1.13)	96.55	-1.12 (-1.21--1.04)	95.92	-1.2 (-1.26--1.14)	90.10	-1.26 (-1.33--1.19)
**Croatia**	30.20	-0.31 (-0.53--0.08)	42.77	0.13 (-0.11-0.37)	20.71	-0.63 (-0.84--0.43)	2.73	-0.89 (-1.09--0.69)
**Cuba**	-36.48	-4.12 (-4.69--3.54)	-35.11	-3.98 (-4.55--3.4)	-37.13	-4.21 (-4.78--3.64)	-40.90	-4.28 (-4.82--3.72)
**Cyprus**	59.84	-1.72 (-1.9--1.55)	80.22	-1.16 (-1.34--0.99)	47.01	-2.06 (-2.22--1.91)	32.84	-2.29 (-2.48--2.1)
**Czech Republic**	-10.45	-2.07 (-2.23--1.92)	-0.58	-1.67 (-1.87--1.47)	-16.36	-2.33 (-2.45--2.21)	-23.81	-2.48 (-2.59--2.36)
**North Korea**	80.45	-0.32 (-0.39--0.25)	78.69	-0.17 (-0.25--0.09)	80.92	-0.46 (-0.54--0.37)	63.31	-0.45 (-0.52--0.38)
**Democratic Republic of the Congo**	94.27	-0.49 (-0.55--0.43)	96.54	-0.43 (-0.51--0.36)	94.31	-0.51 (-0.56--0.46)	92.34	-0.52 (-0.57--0.47)
**Denmark**	49.56	0.21 (-0.01-0.43)	90.61	1.2 (0.94-1.45)	21.40	-0.67 (-0.85--0.49)	13.92	-0.84 (-1--0.67)
**Djibouti**	360.48	0.32 (0.27-0.36)	365.49	0.4 (0.35-0.45)	362.55	0.31 (0.26-0.36)	341.04	0.21 (0.15-0.27)
**Dominica**	-51.42	-3.33 (-3.9--2.76)	-51.34	-3.38 (-3.94--2.81)	-50.84	-3.27 (-3.84--2.69)	-50.45	-3.44 (-4.03--2.85)

**Dominican Republic**	208.97	0.97 (0.44-1.5)	214.52	1.05 (0.53-1.57)	203.51	0.88 (0.36-1.41)	186.00	0.84 (0.29-1.39)
**Ecuador**	110.05	-0.87 (-1.1--0.64)	124.09	-0.65 (-0.86--0.45)	98.37	-1.05 (-1.3--0.81)	79.42	-1.36 (-1.6--1.12)
**Egypt**	125.56	0.27 (0.19-0.35)	139.15	0.43 (0.35-0.5)	115.71	0.15 (0.06-0.24)	114.03	0.1 (0.03-0.18)
**El Salvador**	109.56	-0.36 (-0.56--0.16)	112.56	-0.23 (-0.43--0.03)	107.31	-0.49 (-0.69--0.28)	80.47	-0.68 (-0.87--0.49)
**Equatorial Guinea**	115.64	-0.02 (-0.15-0.1)	128.69	0.18 (0.04-0.31)	110.88	-0.07 (-0.19-0.05)	94.10	-0.53 (-0.69--0.38)
**Eritrea**	223.99	0.75 (0.59-0.91)	236.87	0.85 (0.7-1)	223.49	0.74 (0.57-0.91)	204.57	0.54 (0.38-0.7)
**Estonia**	18.20	-0.32 (-0.45--0.18)	20.09	-0.11 (-0.25-0.04)	17.90	-0.43 (-0.57--0.3)	-3.80	-0.9 (-1.03--0.77)
**Eswatini**	106.55	0.36 (0-0.73)	108.81	0.34 (0.06-0.62)	104.92	0.37 (-0.02-0.76)	107.34	0.41 (-0.03-0.86)
**Ethiopia**	30.72	-1.63 (-1.76--1.49)	33.52	-1.49 (-1.63--1.35)	33.78	-1.56 (-1.69--1.44)	14.58	-2.08 (-2.22--1.94)
**Fiji**	109.77	0.23 (0.04-0.42)	111.11	0.35 (0.14-0.57)	107.39	0.12 (-0.05-0.3)	93.54	0.12 (-0.1-0.34)
**Finland**	21.57	-1.34 (-1.43--1.24)	31.66	-0.9 (-0.97--0.83)	17.10	-1.6 (-1.71--1.48)	1.86	-1.7 (-1.81--1.59)
**France**	-17.93	-2.84 (-2.93--2.75)	-14.23	-2.47 (-2.56--2.39)	-18.55	-3.05 (-3.15--2.95)	-29.69	-3.17 (-3.27--3.07)
**Gabon**	43.96	-0.88 (-0.95--0.82)	50.74	-0.77 (-0.83--0.71)	41.65	-0.9 (-0.98--0.83)	42.57	-1.04 (-1.13--0.95)
**Gambia**	172.27	-0.09 (-0.26-0.08)	171.96	-0.05 (-0.24-0.14)	174.58	-0.09 (-0.25-0.07)	164.19	-0.09 (-0.27-0.1)
**Georgia**	24.43	1.56 (1.17-1.94)	19.04	1.53 (1.15-1.92)	29.73	1.6 (1.23-1.98)	25.49	1.98 (1.57-2.39)
**Germany**	-11.47	-2.01 (-2.24--1.77)	2.85	-1.32 (-1.53--1.1)	-21.59	-2.58 (-2.84--2.32)	-30.43	-2.68 (-2.86--2.5)
**Ghana**	109.44	-0.77 (-0.86--0.68)	113.14	-0.7 (-0.82--0.59)	107.40	-0.79 (-0.87--0.71)	103.63	-0.79 (-0.88--0.7)
**Greece**	59.62	0.25 (-0.2-0.71)	56.86	0.46 (0.01-0.9)	63.59	0.18 (-0.28-0.64)	39.00	0.21 (-0.19-0.61)
**Greenland**	50.17	-1.46 (-1.71--1.21)	52.96	-1.27 (-1.49--1.05)	49.07	-1.54 (-1.81--1.28)	30.25	-1.72 (-1.94--1.49)
**Grenada**	-48.89	-3.98 (-4.6--3.34)	-44.32	-3.9 (-4.53--3.27)	-51.47	-4 (-4.62--3.38)	-46.09	-4.11 (-4.77--3.44)
**Guam**	25.34	-3.33 (-3.51--3.14)	26.88	-2.98 (-3.14--2.82)	24.25	-3.6 (-3.82--3.37)	16.40	-3.1 (-3.27--2.93)
**Guatemala**	18.00	-4.53 (-4.89--4.16)	21.34	-4.28 (-4.64--3.92)	16.71	-4.65 (-5.02--4.27)	6.08	-4.61 (-4.98--4.24)
**Guinea**	34.23	-0.52 (-0.62--0.42)	37.07	-0.47 (-0.58--0.36)	33.19	-0.54 (-0.63--0.46)	36.34	-0.5 (-0.6--0.39)
**Guinea-Bissau**	27.36	-0.98 (-1.1--0.87)	32.46	-0.89 (-1.02--0.76)	25.30	-1 (-1.11--0.9)	28.84	-1.04 (-1.15--0.94)
**Guyana**	-36.31	-3.46 (-3.96--2.95)	-34.59	-3.4 (-3.9--2.88)	-37.22	-3.49 (-3.98--2.99)	-36.54	-3.41 (-3.9--2.93)
**Haiti**	18.09	-1.96 (-2.21--1.71)	20.96	-1.88 (-2.15--1.62)	17.49	-1.98 (-2.21--1.74)	14.88	-2.07 (-2.32--1.82)
**Honduras**	277.25	1.11 (0.93-1.29)	275.22	1.1 (0.93-1.28)	276.94	1.14 (0.95-1.33)	241.00	0.81 (0.64-0.97)
**Hungary**	-35.94	-2.76 (-2.88--2.65)	-35.58	-2.66 (-2.77--2.55)	-34.70	-2.75 (-2.87--2.63)	-39.24	-2.7 (-2.84--2.57)
**Iceland**	15.46	-1.94 (-2.04--1.85)	19.14	-1.71 (-1.79--1.62)	13.27	-2.06 (-2.18--1.93)	3.61	-2.24 (-2.33--2.16)
**India**	256.09	1.15 (1-1.29)	260.24	1.35 (1.22-1.47)	255.70	1.03 (0.87-1.19)	226.75	1.12 (0.98-1.26)
**Indonesia**	102.57	-0.1 (-0.14--0.06)	108.98	-0.01 (-0.05-0.03)	98.78	-0.17 (-0.21--0.13)	80.71	-0.49 (-0.52--0.47)

**Iran**	172.03	-0.23 (-0.38--0.07)	183.10	-0.02 (-0.18-0.15)	164.76	-0.37 (-0.54--0.21)	132.70	-0.47 (-0.63--0.31)
**Iraq**	175.87	-0.15 (-0.3-0.01)	203.47	0.15 (-0.04-0.35)	163.12	-0.25 (-0.39--0.11)	167.52	-0.45 (-0.56--0.35)
**Ireland**	16.89	-1.83 (-2.14--1.52)	29.75	-1.33 (-1.61--1.05)	10.48	-2.14 (-2.46--1.81)	1.03	-2.36 (-2.67--2.04)
**Israel**	11.92	-3.08 (-3.29--2.87)	19.17	-2.7 (-2.92--2.47)	7.17	-3.3 (-3.49--3.11)	-2.53	-3.4 (-3.59--3.2)
**Italy**	31.54	-1.1 (-1.24--0.97)	39.36	-0.72 (-0.88--0.56)	26.05	-1.38 (-1.49--1.26)	4.47	-1.6 (-1.7--1.5)
**Jamaica**	17.95	-0.99 (-1.31--0.66)	22.07	-0.87 (-1.17--0.55)	16.09	-1.06 (-1.4--0.73)	23.82	-0.9 (-1.23--0.57)
**Japan**	69.12	-1.48 (-1.53--1.43)	72.32	-1.02 (-1.1--0.94)	68.04	-1.81 (-1.85--1.76)	17.83	-2.17 (-2.22--2.12)
**Jordan**	273.14	-0.96 (-1.05--0.87)	303.03	-0.66 (-0.74--0.58)	253.87	-1.13 (-1.22--1.03)	232.51	-1.43 (-1.55--1.31)
**Kazakhstan**	19.63	-0.79 (-1.03--0.55)	19.97	-0.76 (-0.94--0.57)	19.62	-0.8 (-1.07--0.52)	18.73	-0.91 (-1.21--0.61)
**Kenya**	190.91	0.59 (0.49-0.68)	198.03	0.61 (0.51-0.71)	215.72	1.12 (0.95-1.29)	220.94	0.98 (0.81-1.15)
**Kiribati**	61.42	-0.31 (-0.36--0.27)	67.58	-0.27 (-0.32--0.22)	56.80	-0.34 (-0.39--0.29)	57.31	-0.51 (-0.57--0.45)
**Kuwait**	130.34	-1.81 (-2.03--1.58)	144.09	-1.58 (-1.83--1.32)	124.54	-1.92 (-2.13--1.71)	106.82	-2.16 (-2.34--1.97)
**Kyrgyzstan**	29.42	-0.35 (-0.53--0.18)	32.74	-0.36 (-0.5--0.22)	28.86	-0.29 (-0.5--0.07)	36.04	-0.44 (-0.62--0.25)
**Laos**	38.47	-1.53 (-1.69--1.36)	47.20	-1.35 (-1.52--1.18)	34.64	-1.6 (-1.76--1.44)	25.95	-1.93 (-2.09--1.77)
**Latvia**	1.48	-0.52 (-0.86--0.17)	0.97	-0.4 (-0.72--0.08)	2.62	-0.57 (-0.92--0.21)	-9.72	-0.74 (-1.13--0.35)
**Lebanon**	141.01	0.26 (0.13-0.4)	161.08	0.7 (0.56-0.83)	125.83	-0.08 (-0.22-0.06)	93.74	-0.26 (-0.38--0.14)
**Lesotho**	81.32	1.9 (1.63-2.17)	83.09	1.85 (1.6-2.1)	79.23	1.89 (1.62-2.17)	92.75	2.09 (1.78-2.4)
**Liberia**	19.38	-1.18 (-1.37--0.98)	25.70	-1.08 (-1.29--0.87)	16.45	-1.22 (-1.41--1.03)	25.03	-1.25 (-1.46--1.03)
**Libya**	147.95	-0.12 (-0.3-0.06)	157.26	-0.02 (-0.2-0.17)	140.97	-0.22 (-0.39--0.05)	145.80	-0.25 (-0.41--0.08)
**Lithuania**	14.23	-0.76 (-1.08--0.44)	14.53	-0.64 (-0.95--0.33)	13.91	-0.84 (-1.17--0.52)	-2.69	-1.07 (-1.41--0.73)
**Luxembourg**	-16.24	-2.89 (-2.97--2.8)	-8.92	-2.48 (-2.57--2.4)	-16.35	-2.99 (-3.09--2.89)	-23.53	-3.12 (-3.22--3.01)
**Madagascar**	113.12	0.08 (-0.02-0.17)	120.00	0.08 (0-0.16)	109.41	0.08 (-0.01-0.18)	120.26	0 (-0.09-0.09)
**Malawi**	74.63	-0.29 (-0.37--0.2)	78.07	-0.14 (-0.2--0.08)	75.69	-0.28 (-0.36--0.2)	69.56	-0.41 (-0.52--0.31)
**Malaysia**	182.97	-0.3 (-0.42--0.18)	210.09	0.07 (-0.05-0.2)	163.24	-0.57 (-0.7--0.44)	145.37	-0.74 (-0.89--0.58)
**Maldives**	116.53	-1.92 (-2.17--1.67)	150.29	-1.3 (-1.57--1.03)	98.11	-2.29 (-2.53--2.06)	62.91	-2.85 (-3.13--2.57)
**Mali**	74.24	-0.51 (-0.61--0.41)	79.12	-0.4 (-0.5--0.3)	73.76	-0.51 (-0.61--0.42)	69.87	-0.61 (-0.71--0.5)
**Malta**	21.19	-2.25 (-2.33--2.17)	26.79	-1.88 (-1.95--1.81)	15.79	-2.5 (-2.58--2.42)	1.91	-2.54 (-2.61--2.47)
**Marshall Islands**	80.10	-0.55 (-0.61--0.5)	95.01	-0.4 (-0.45--0.34)	69.55	-0.67 (-0.72--0.62)	86.84	-0.61 (-0.68--0.55)
**Mauritania**	18.86	-1.74 (-1.95--1.54)	23.43	-1.6 (-1.82--1.39)	17.98	-1.77 (-1.96--1.58)	12.85	-1.91 (-2.11--1.7)
**Mauritius**	80.66	-1.02 (-1.34--0.7)	92.07	-0.72 (-1.06--0.39)	73.59	-1.22 (-1.53--0.92)	61.42	-1.25 (-1.57--0.94)
**Mexico**	60.84	-2.34 (-2.52--2.17)	66.82	-2.18 (-2.35--2)	56.70	-2.46 (-2.62--2.29)	48.68	-2.46 (-2.64--2.29)

**Micronesia (Federated States of)**	31.71	-0.52 (-0.61--0.44)	41.75	-0.35 (-0.42--0.27)	24.88	-0.66 (-0.76--0.57)	28.19	-0.73 (-0.82--0.64)
**Monaco**	-27.62	-2.09 (-2.46--1.71)	-28.39	-1.91 (-2.3--1.53)	-26.83	-2.22 (-2.58--1.86)	-29.31	-2.23 (-2.58--1.87)
**Mongolia**	84.07	-1.01 (-1.2--0.82)	92.53	-0.94 (-1.1--0.78)	80.83	-0.98 (-1.2--0.77)	94.02	-1.15 (-1.36--0.94)
**Montenegro**	56.57	-0.02 (-0.12-0.07)	49.83	-0.14 (-0.2--0.07)	62.30	0.09 (-0.04-0.21)	50.09	-0.11 (-0.29-0.06)
**Morocco**	133.35	0.09 (0.01-0.16)	143.82	0.23 (0.15-0.31)	128.88	0.03 (-0.04-0.09)	120.95	-0.08 (-0.13--0.04)
**Mozambique**	136.11	1.23 (1.09-1.38)	141.94	1.31 (1.15-1.47)	132.38	1.17 (1.04-1.31)	135.48	1.24 (1.07-1.4)
**Myanmar**	40.02	-1.27 (-1.34--1.21)	46.49	-1.09 (-1.16--1.02)	36.56	-1.37 (-1.43--1.31)	22.01	-1.73 (-1.8--1.67)
**Namibia**	96.50	-0.09 (-0.29-0.11)	104.08	0.06 (-0.1-0.23)	93.68	-0.16 (-0.37-0.06)	87.08	-0.32 (-0.56--0.08)
**Nauru**	-4.34	-0.65 (-0.77--0.54)	3.38	-0.53 (-0.63--0.44)	-11.03	-0.79 (-0.92--0.66)	-4.87	-0.76 (-0.93--0.59)
**Nepal**	241.56	1.46 (1.35-1.58)	242.77	1.53 (1.42-1.64)	242.83	1.45 (1.34-1.55)	199.67	1.15 (1.03-1.27)
**Netherlands**	14.03	-1.27 (-1.51--1.03)	18.64	-0.96 (-1.23--0.69)	11.97	-1.44 (-1.65--1.23)	4.12	-1.55 (-1.74--1.35)
**New Zealand**	79.76	-0.26 (-0.37--0.14)	108.76	0.34 (0.23-0.45)	58.11	-0.78 (-0.89--0.66)	44.25	-0.9 (-1--0.79)
**Nicaragua**	161.09	-0.82 (-1.27--0.35)	168.56	-0.68 (-1.07--0.28)	158.74	-0.86 (-1.39--0.32)	128.61	-1.23 (-1.62--0.84)
**Niger**	117.51	-0.9 (-1.03--0.77)	121.90	-0.82 (-0.96--0.68)	116.70	-0.92 (-1.04--0.8)	112.05	-0.99 (-1.13--0.85)
**Nigeria**	91.88	0.26 (0.16-0.36)	97.37	0.33 (0.21-0.45)	90.53	0.26 (0.16-0.35)	92.53	0.14 (0.05-0.22)
**Niue**	-19.52	-0.79 (-0.85--0.74)	-12.31	-0.57 (-0.63--0.51)	-26.40	-1.06 (-1.12--1)	-22.32	-1.12 (-1.18--1.05)
**Macedonia**	45.68	-1.01 (-1.44--0.58)	44.54	-1.03 (-1.43--0.62)	47.27	-0.96 (-1.41--0.51)	32.95	-1.29 (-1.73--0.85)
**Northern Mariana Islands**	134.52	-0.87 (-1.23--0.52)	135.64	-0.69 (-1.09--0.28)	134.05	-1.02 (-1.34--0.7)	106.26	-1.06 (-1.38--0.73)
**Norway**	32.97	-0.09 (-0.23-0.05)	75.34	1.04 (0.88-1.2)	3.77	-1.17 (-1.31--1.03)	-2.50	-1.41 (-1.55--1.27)
**Oman**	93.68	-0.43 (-0.59--0.26)	117.58	-0.21 (-0.39--0.04)	77.74	-0.57 (-0.74--0.4)	77.46	-0.95 (-1.18--0.73)
**Pakistan**	136.98	0.6 (0.33-0.88)	147.85	0.65 (0.4-0.91)	129.51	0.57 (0.29-0.86)	145.77	0.49 (0.19-0.8)
**Palau**	98.10	-0.31 (-0.34--0.28)	113.50	-0.15 (-0.19--0.11)	84.28	-0.51 (-0.55--0.47)	93.02	-0.54 (-0.59--0.5)
**Palestine**	147.38	-0.21 (-0.54-0.11)	164.22	-0.08 (-0.45-0.29)	137.20	-0.28 (-0.58-0.01)	147.87	-0.37 (-0.68--0.07)
**Panama**	45.54	-2.23 (-2.49--1.96)	50.29	-2.07 (-2.35--1.79)	43.04	-2.32 (-2.57--2.06)	34.15	-2.27 (-2.55--2)
**Papua New Guinea**	148.40	-0.11 (-0.13--0.09)	160.18	0 (-0.02-0.02)	142.83	-0.17 (-0.19--0.15)	146.75	-0.17 (-0.19--0.15)
**Paraguay**	116.92	-0.86 (-1.01--0.71)	124.73	-0.75 (-0.91--0.59)	110.53	-0.96 (-1.1--0.82)	108.19	-0.96 (-1.1--0.82)
**Peru**	140.21	-0.58 (-0.8--0.37)	161.32	-0.18 (-0.38-0.02)	123.95	-0.91 (-1.12--0.69)	98.20	-1.08 (-1.31--0.86)
**Philippines**	111.46	-0.93 (-1.12--0.73)	117.54	-0.81 (-1--0.62)	105.71	-1.05 (-1.25--0.85)	99.39	-1.05 (-1.23--0.86)
**Poland**	-1.88	-2.05 (-2.16--1.93)	-1.78	-1.9 (-2.01--1.79)	-0.15	-2.07 (-2.18--1.97)	-9.70	-2.09 (-2.16--2.01)

**Portugal**	31.60	-1.29 (-1.37--1.21)	57.22	-0.43 (-0.5--0.35)	14.43	-1.96 (-2.06--1.86)	-2.24	-1.98 (-2.09--1.88)
**Puerto Rico**	8.44	-2.49 (-2.65--2.32)	12.23	-2.17 (-2.32--2.02)	7.08	-2.69 (-2.86--2.52)	-4.86	-2.68 (-2.86--2.51)
**Qatar**	369.31	-1.38 (-1.6--1.16)	454.82	-0.86 (-1.09--0.63)	312.69	-1.66 (-1.88--1.45)	321.65	-2.14 (-2.4--1.88)
**South Korea**	138.74	-1.26 (-1.36--1.16)	177.74	-0.54 (-0.65--0.43)	110.81	-1.81 (-1.9--1.71)	50.73	-2.59 (-2.7--2.48)
**Moldova**	-37.37	-2.7 (-2.98--2.42)	-38.59	-2.62 (-2.92--2.32)	-35.83	-2.72 (-2.99--2.46)	-42.40	-2.77 (-3.07--2.47)
**Romania**	-17.22	-1.37 (-1.66--1.08)	-18.76	-1.29 (-1.61--0.97)	-15.51	-1.38 (-1.66--1.11)	-26.01	-1.5 (-1.79--1.22)
**Russia**	7.60	-1.06 (-1.28--0.83)	18.68	-0.53 (-0.7--0.36)	-1.05	-1.47 (-1.74--1.21)	-7.28	-1.57 (-1.87--1.27)
**Rwanda**	64.58	-1.11 (-1.31--0.91)	73.93	-0.85 (-1.05--0.65)	63.57	-1.11 (-1.31--0.91)	54.43	-1.57 (-1.82--1.32)
**Saint Kitts and Nevis**	-54.04	-4.43 (-5.02--3.84)	-51.08	-4.52 (-5.11--3.92)	-56.20	-4.4 (-4.99--3.81)	-50.90	-4.77 (-5.38--4.15)
**Saint Lucia**	-20.35	-4.38 (-5.12--3.64)	-16.57	-4.18 (-4.91--3.45)	-21.90	-4.49 (-5.23--3.75)	-23.25	-4.45 (-5.19--3.7)
**Saint Vincent and the Grenadines**	-26.07	-3.18 (-3.76--2.59)	-24.71	-3.16 (-3.73--2.59)	-26.54	-3.17 (-3.77--2.57)	-25.07	-3.24 (-3.8--2.67)
**Samoa**	43.63	-0.66 (-0.7--0.62)	50.90	-0.5 (-0.53--0.47)	37.82	-0.8 (-0.86--0.74)	37.66	-0.78 (-0.82--0.73)
**San Marino**	51.64	-0.98 (-1.15--0.82)	50.46	-0.82 (-1--0.64)	61.29	-0.85 (-0.97--0.74)	42.49	-0.9 (-1.02--0.77)
**Sao Tome and Principe**	81.18	0.45 (0.35-0.55)	87.65	0.5 (0.41-0.6)	76.70	0.41 (0.32-0.51)	84.28	0.32 (0.18-0.45)
**Saudi Arabia**	249.12	0.61 (0.21-1.01)	312.99	1.01 (0.67-1.36)	167.76	-0.3 (-0.66-0.06)	213.05	-0.19 (-0.51-0.14)
**Senegal**	80.18	-0.74 (-0.94--0.55)	83.92	-0.66 (-0.88--0.44)	78.86	-0.78 (-0.96--0.59)	77.60	-0.78 (-1--0.56)
**Serbia**	30.82	-0.56 (-0.69--0.42)	25.75	-0.57 (-0.71--0.44)	34.86	-0.52 (-0.65--0.39)	15.76	-0.78 (-0.93--0.62)
**Seychelles**	61.41	-0.98 (-1.22--0.75)	81.29	-0.72 (-0.97--0.48)	47.75	-1.18 (-1.41--0.95)	55.51	-1.37 (-1.63--1.11)
**Sierra Leone**	43.50	-0.64 (-0.76--0.52)	49.40	-0.58 (-0.72--0.43)	39.70	-0.69 (-0.8--0.58)	50.12	-0.61 (-0.74--0.49)
**Singapore**	171.93	-1.21 (-1.51--0.92)	211.19	-0.58 (-0.89--0.27)	144.84	-1.68 (-1.96--1.4)	111.12	-2 (-2.29--1.7)
**Slovakia**	33.28	-0.6 (-0.71--0.49)	42.53	-0.32 (-0.47--0.18)	26.40	-0.83 (-0.94--0.72)	14.58	-1.11 (-1.21--1.02)
**Slovenia**	53.55	-0.52 (-0.67--0.38)	83.40	0.31 (0.16-0.46)	32.00	-1.2 (-1.36--1.05)	11.36	-1.48 (-1.63--1.33)
**Solomon Islands**	122.95	-0.08 (-0.14--0.01)	135.81	0.05 (-0.03-0.13)	114.42	-0.17 (-0.22--0.11)	115.46	-0.23 (-0.29--0.17)
**Somalia**	169.08	0.41 (0.32-0.49)	169.61	0.44 (0.35-0.53)	174.70	0.48 (0.39-0.56)	166.71	0.37 (0.29-0.45)
**South Africa**	106.55	-0.08 (-0.4-0.23)	106.67	-0.08 (-0.35-0.18)	107.83	-0.08 (-0.42-0.27)	95.35	-0.18 (-0.5-0.14)
**South Sudan**	45.06	-0.16 (-0.24--0.08)	49.79	-0.11 (-0.2--0.02)	44.13	-0.15 (-0.22--0.08)	49.61	-0.22 (-0.3--0.14)
**Spain**	21.39	-1.7 (-1.86--1.55)	37.96	-1.12 (-1.26--0.99)	8.35	-2.22 (-2.38--2.05)	-8.49	-2.36 (-2.51--2.2)
**Sri Lanka**	27.06	-3.26 (-3.85--2.67)	42.21	-2.8 (-3.4--2.2)	16.21	-3.62 (-4.2--3.03)	9.28	-3.67 (-4.3--3.05)
**Sudan**	83.42	-0.21 (-0.32--0.11)	93.32	-0.09 (-0.2-0.02)	76.95	-0.29 (-0.38--0.19)	75.25	-0.44 (-0.53--0.35)

**Suriname**	-0.52	-3.03 (-3.64--2.42)	1.21	-2.98 (-3.6--2.35)	-2.22	-3.08 (-3.68--2.48)	-3.94	-3.15 (-3.75--2.55)
**Sweden**	-37.01	-2.91 (-3.2--2.61)	-48.83	-3.44 (-3.85--3.02)	-20.08	-2.27 (-2.46--2.08)	-26.17	-2.44 (-2.63--2.25)
**Switzerland**	39.22	-1.09 (-1.41--0.76)	41.70	-0.91 (-1.22--0.6)	41.23	-1.12 (-1.47--0.77)	27.15	-1.28 (-1.61--0.95)
**Syria**	106.30	-0.76 (-0.99--0.53)	120.20	-0.51 (-0.72--0.3)	96.14	-0.94 (-1.17--0.71)	90.53	-1.08 (-1.31--0.84)
**Taiwan**	166.66	-0.55 (-1.27-0.18)	188.59	-0.07 (-0.9-0.76)	150.35	-0.94 (-1.57--0.31)	106.56	-1.24 (-1.87--0.6)
**Tajikistan**	69.37	0.5 (0.32-0.68)	71.40	0.31 (0.11-0.51)	68.71	0.66 (0.51-0.82)	81.84	0.18 (-0.01-0.38)
**Thailand**	233.43	0.37 (0.26-0.47)	268.66	0.93 (0.84-1.02)	210.81	-0.04 (-0.16-0.08)	175.67	-0.06 (-0.17-0.05)
**Timor-Leste**	164.42	-0.32 (-0.6--0.03)	162.23	-0.17 (-0.46-0.13)	166.22	-0.41 (-0.68--0.13)	119.61	-0.69 (-0.99--0.39)
**Togo**	117.66	-1.01 (-1.13--0.9)	123.43	-0.96 (-1.1--0.81)	115.33	-1.02 (-1.13--0.91)	121.23	-1.02 (-1.13--0.92)
**Tokelau**	-26.64	-1.04 (-1.07--1)	-19.47	-0.8 (-0.84--0.77)	-31.49	-1.22 (-1.26--1.19)	-29.80	-1.35 (-1.38--1.31)
**Tonga**	39.44	-0.27 (-0.42--0.13)	41.34	-0.12 (-0.27-0.02)	38.41	-0.39 (-0.53--0.25)	26.76	-0.43 (-0.56--0.3)
**Trinidad and Tobago**	-26.88	-4.41 (-5.06--3.75)	-24.74	-4.23 (-4.89--3.57)	-27.35	-4.48 (-5.13--3.83)	-30.21	-4.51 (-5.16--3.86)
**Tunisia**	145.04	-0.22 (-0.26--0.18)	157.28	0.04 (0-0.07)	135.58	-0.41 (-0.46--0.37)	118.86	-0.47 (-0.51--0.42)
**Turkey**	59.31	-1.58 (-1.68--1.48)	71.51	-1.26 (-1.35--1.16)	50.56	-1.83 (-1.94--1.71)	29.21	-2.3 (-2.4--2.19)
**Turkmenistan**	-14.87	-3.18 (-4.04--2.31)	-14.23	-3.09 (-4--2.16)	-14.78	-3.21 (-4.04--2.38)	-12.83	-3.09 (-4.01--2.17)
**Tuvalu**	12.66	-1.09 (-1.16--1.02)	16.85	-0.93 (-0.99--0.87)	9.96	-1.21 (-1.29--1.14)	2.50	-1.32 (-1.39--1.25)
**Uganda**	166.33	0.73 (0.58-0.89)	182.40	0.94 (0.79-1.09)	159.49	0.69 (0.55-0.83)	170.65	0.68 (0.49-0.86)
**Ukraine**	14.19	0.01 (-0.16-0.18)	20.58	0.37 (0.22-0.52)	8.63	-0.28 (-0.46--0.1)	8.30	-0.12 (-0.32-0.08)
**United Arab Emirates**	637.13	-1.56 (-2.07--1.05)	718.05	-1.37 (-1.83--0.91)	576.38	-1.7 (-2.24--1.15)	662.49	-1.55 (-1.98--1.12)
**UK**	23.90	-0.31 (-0.53--0.09)	43.26	0.27 (0.08-0.45)	6.26	-0.93 (-1.18--0.68)	-3.49	-1.12 (-1.35--0.88)
**Tanzania**	135.16	0.32 (0.23-0.41)	140.77	0.44 (0.32-0.56)	134.45	0.3 (0.21-0.39)	131.62	0.26 (0.16-0.36)
**USA**	34.62	-0.87 (-0.91--0.84)	51.03	-0.44 (-0.51--0.37)	20.08	-1.29 (-1.36--1.23)	18.62	-1.29 (-1.35--1.22)
**United States Virgin Islands**	51.37	-1.34 (-1.77--0.91)	48.88	-1.24 (-1.66--0.81)	52.54	-1.42 (-1.85--0.99)	32.65	-1.47 (-1.92--1.02)
**Uruguay**	6.17	-1.07 (-1.15--0.99)	8.61	-0.86 (-0.94--0.78)	6.21	-1.19 (-1.26--1.12)	-3.20	-1.21 (-1.27--1.14)
**Uzbekistan**	100.58	0.83 (0.48-1.17)	110.58	0.68 (0.42-0.94)	92.69	0.92 (0.53-1.3)	131.43	0.7 (0.38-1.02)
**Vanuatu**	161.66	-0.35 (-0.5--0.21)	168.73	-0.25 (-0.42--0.09)	156.66	-0.43 (-0.57--0.29)	154.35	-0.4 (-0.57--0.24)
**Venezuela**	57.52	-2.63 (-2.97--2.3)	63.50	-2.45 (-2.77--2.13)	53.97	-2.76 (-3.1--2.41)	45.49	-2.83 (-3.18--2.48)
**Vietnam**	197.80	1.36 (1.16-1.55)	242.43	1.84 (1.63-2.06)	166.44	0.96 (0.79-1.14)	176.77	1.05 (0.84-1.26)
**Yemen**	155.23	-0.18 (-0.26--0.11)	163.80	-0.04 (-0.12-0.05)	151.73	-0.24 (-0.31--0.17)	148.94	-0.31 (-0.38--0.24)
**Zambia**	135.77	-0.03 (-0.11-0.05)	146.65	0.17 (0.13-0.21)	131.06	-0.07 (-0.16-0.02)	131.94	-0.18 (-0.3--0.06)

**Zimbabwe**	63.60	0.22 (0-0.44)	62.36	0.17 (-0.04-0.38)	63.73	0.4 (0.14-0.67)	73.34	0.63 (0.33-0.93)

Supplementary Table S8. The percentage change of GBTC cases from 1990 to 2019 by SDI and age

Socio-demographic index (SDI)	Incidence		Prevalence		Deaths		DALYs	
	Number in 2019 ×10³	Percentage change in Absolute number (%)	Number in 2019 ×10³	Percentage change in Absolute number (%)	Number in 2019 ×10³	Percentage change in Absolute number (%)	Number in 2019 ×10³	Percentage change in Absolute number (%)
**Global**								
20-24 years	0.26	10.25	0.51	18.40	0.14	1.32	9.26	1.36
25-29 years	0.48	20.41	0.87	28.38	0.27	11.73	16.98	11.80
30-34 years	1.07	41.97	1.83	50.99	0.67	31.98	38.08	32.07
35-39 years	2.04	36.34	3.40	45.74	1.31	26.30	68.25	26.40
40-44 years	3.70	52.74	6.20	61.41	2.53	43.99	119.42	44.12
45-49 years	6.18	66.76	8.95	71.16	4.84	62.32	204.18	62.33
50-54 years	10.94	70.29	15.56	74.94	9.00	66.78	336.98	66.79
55-59 years	16.24	63.98	23.46	71.35	13.06	59.10	427.78	59.14
60-64 years	22.00	60.82	29.79	67.45	18.45	56.88	519.26	56.92
65-69 years	26.95	67.18	35.79	74.48	22.58	63.31	533.95	63.28
70-74 years	28.78	85.03	36.51	95.58	24.99	79.21	483.89	79.28
75-79 years	27.47	67.41	33.88	81.24	24.62	61.75	377.58	61.57
80-84 years	24.72	106.62	30.60	127.55	22.39	99.35	264.60	98.87
85-89 years	18.34	178.49	20.86	212.46	16.47	167.30	149.16	166.48
90-94 years	7.67	326.30	6.87	377.55	7.89	304.43	55.34	302.27
>95 years	2.38	594.78	1.24	602.75	3.23	562.14	16.76	541.80
Early-onset	13.72	52.36	21.77	58.55	9.76	46.47	456.17	44.45
Later-onset	185.49	87.78	234.57	95.49	162.68	84.46	3165.31	72.24
Total	199.21	84.82	256.34	91.69	172.44	81.79	3621.47	68.16
**High SDI**								
20-24 years	0.03	-17.65	0.08	-7.32	0.01	-38.46	0.55	-36.84
25-29 years	0.05	-26.87	0.12	-17.33	0.02	-41.38	1.05	-41.29

30-34 years	0.13	-16.46	0.32	-6.78	0.05	-31.08	2.91	-31.07
35-39 years	0.28	-17.92	0.67	-7.86	0.11	-32.14	5.97	-32.02
40-44 years	0.53	-23.00	1.23	-12.91	0.24	-35.29	11.48	-34.97
45-49 years	0.88	-16.35	1.64	-5.79	0.52	-26.69	21.97	-26.62
50-54 years	1.55	-15.27	2.76	-4.27	1.03	-24.63	38.69	-24.52
55-59 years	2.90	-6.94	5.36	8.23	1.85	-19.12	60.78	-18.93
60-64 years	4.44	-1.77	7.61	12.95	3.02	-13.04	85.11	-12.83
65-69 years	6.59	2.92	11.10	18.26	4.43	-8.33	105.03	-8.11
70-74 years	8.76	32.65	13.74	50.30	6.25	20.16	121.28	20.43
75-79 years	10.32	28.03	15.56	49.30	7.72	15.74	118.61	15.77
80-84 years	10.90	63.64	16.09	91.48	8.42	50.48	99.56	50.17
85-89 years	9.76	144.27	12.68	181.04	7.69	131.72	69.69	130.76
90-94 years	4.93	315.77	4.73	366.27	4.73	299.16	33.11	296.42
>95 years	1.79	665.38	0.94	674.38	2.33	646.47	12.03	620.98
Early-onset	1.90	-18.86	4.06	-8.87	0.95	-30.38	43.93	-30.55
Later-onset	61.94	45.38	90.56	57.31	47.46	38.64	743.87	14.82
Total	63.84	42.04	94.62	52.56	48.41	35.99	787.80	10.79
**High-middle SDI**								
20-24 years	0.04	-25.86	0.09	-14.15	0.02	-36.36	1.40	-37.73
25-29 years	0.10	-5.00	0.18	7.60	0.05	-19.35	3.11	-18.78
30-34 years	0.26	24.15	0.46	41.36	0.15	6.38	8.57	6.34
35-39 years	0.47	12.71	0.82	30.72	0.29	-4.36	14.83	-4.26
40-44 years	0.88	35.79	1.57	56.80	0.57	17.36	26.82	17.80
45-49 years	1.44	52.60	2.12	63.33	1.09	42.08	46.18	42.17
50-54 years	2.63	49.54	3.79	58.60	2.13	42.43	79.55	42.50
55-59 years	4.16	47.85	6.07	59.33	3.30	40.02	108.17	40.05
60-64 years	5.81	40.95	7.88	50.03	4.85	35.00	136.31	35.06
65-69 years	7.36	57.55	9.73	70.73	6.14	48.91	145.20	48.91
70-74 years	7.66	79.36	9.57	96.23	6.66	68.33	128.91	68.42
75-79 years	6.97	48.55	8.31	66.89	6.34	38.90	97.11	38.75
80-84 years	6.43	103.48	7.60	136.27	5.96	87.44	70.29	86.85

85-89 years	4.62	190.98	4.93	248.83	4.31	162.40	38.98	161.89
90-94 years	1.61	312.53	1.37	385.16	1.71	269.48	12.00	268.33
>95 years	0.35	410.29	0.18	414.29	0.49	368.27	2.59	361.61
Early-onset	3.19	34.16	5.24	48.64	2.17	21.25	100.91	18.90
Later-onset	47.59	72.86	59.44	85.98	41.87	64.39	819.11	53.29
Total	50.77	69.80	64.67	82.28	44.04	61.57	920.02	48.58
**Middle SDI**								
20-24 years	0.08	-11.63	0.15	-0.68	0.04	-24.53	2.70	-23.76
25-29 years	0.15	8.21	0.26	23.47	0.08	-5.62	5.19	-5.76
30-34 years	0.32	41.67	0.53	63.00	0.21	22.62	11.74	22.90
35-39 years	0.62	40.64	0.99	64.29	0.41	19.82	21.10	20.18
40-44 years	1.13	83.79	1.84	119.19	0.79	56.55	37.20	56.94
45-49 years	1.88	110.07	2.60	126.37	1.51	95.33	63.53	95.47
50-54 years	3.37	125.74	4.62	139.65	2.84	114.77	106.10	114.89
55-59 years	4.72	121.85	6.43	139.91	3.95	109.50	129.27	109.53
60-64 years	6.11	129.07	7.72	146.49	5.37	117.21	150.84	117.25
65-69 years	6.97	151.75	8.36	175.28	6.24	136.02	147.31	135.94
70-74 years	6.77	159.47	7.54	185.10	6.40	142.94	123.84	142.94
75-79 years	5.83	174.61	6.01	206.89	5.86	155.63	89.73	155.08
80-84 years	4.45	241.52	4.36	284.83	4.67	215.55	55.10	215.14
85-89 years	2.54	286.74	2.18	334.66	2.77	255.58	25.07	255.54
90-94 years	0.73	400.68	0.52	451.06	0.92	360.30	6.43	358.92
>95 years	0.16	519.23	0.08	530.77	0.27	476.09	1.38	462.20
Early-onset	3.19	34.16	6.37	94.42	3.03	57.57	141.47	53.16
Later-onset	4.17	74.05	47.83	179.31	39.27	149.34	835.07	135.05
Total	41.64	161.63	54.20	165.67	42.30	139.38	976.54	118.15
**Low-middle**								
20-24 years	0.08	77.78	0.14	86.84	0.05	68.97	3.25	68.05
25-29 years	0.14	86.30	0.22	97.35	0.09	78.00	5.51	76.52
30-34 years	0.26	114.75	0.39	128.24	0.19	103.23	10.78	103.88
35-39 years	0.49	123.39	0.69	137.72	0.37	110.34	19.04	110.16

40-44 years	0.85	144.38	1.17	161.61	0.68	130.51	32.04	130.60
45-49 years	1.48	143.00	1.92	151.11	1.26	136.26	53.28	136.22
50-54 years	2.57	159.70	3.34	166.88	2.27	154.94	84.91	154.87
55-59 years	3.39	149.04	4.27	157.26	2.99	143.60	97.85	143.68
60-64 years	4.28	142.21	5.02	150.42	3.95	137.46	111.03	137.41
65-69 years	4.59	172.46	5.05	184.50	4.37	166.30	103.27	166.08
70-74 years	4.27	177.32	4.34	190.37	4.30	171.06	83.24	170.94
75-79 years	3.36	191.23	3.12	207.29	3.61	183.66	55.36	182.84
80-84 years	2.34	260.90	2.04	279.00	2.65	250.93	31.31	249.60
85-89 years	1.15	320.96	0.87	337.00	1.36	309.94	12.34	309.22
90-94 years	0.32	442.37	0.21	459.46	0.43	429.27	3.05	429.74
>95 years	0.07	475.00	0.04	483.33	0.12	495.00	0.62	472.48
Early-onset	3.29	132.86	4.53	143.58	2.64	124.23	123.89	121.84
Later-onset	26.32	177.57	28.29	183.47	26.05	175.11	582.98	162.60
Total	29.60	171.77	32.82	177.20	28.68	169.46	706.87	154.41
**Low SDI**								
20-24 years	0.03	175.00	0.06	175.00	0.02	150.00	1.36	151.02
25-29 years	0.05	127.27	0.08	146.88	0.03	126.67	2.11	124.42
30-34 years	0.10	150.00	0.13	162.75	0.07	136.67	4.06	139.55
35-39 years	0.18	143.84	0.24	158.06	0.14	137.29	7.27	136.37
40-44 years	0.30	157.76	0.39	172.22	0.25	146.08	11.82	146.71
45-49 years	0.52	137.61	0.66	144.44	0.45	132.82	19.13	132.21
50-54 years	0.82	130.25	1.05	137.10	0.74	126.77	27.59	126.78
55-59 years	1.07	120.37	1.32	127.19	0.96	115.92	31.52	115.91
60-64 years	1.35	127.06	1.55	134.95	1.27	123.77	35.75	123.73
65-69 years	1.44	144.05	1.53	154.82	1.39	139.11	32.95	138.89
70-74 years	1.33	149.25	1.30	161.12	1.37	144.80	26.44	144.40
75-79 years	0.98	165.31	0.87	178.85	1.08	161.20	16.64	160.30
80-84 years	0.60	219.89	0.50	234.46	0.70	214.93	8.25	214.25
85-89 years	0.27	287.14	0.20	302.04	0.33	282.76	3.03	282.55
90-94 years	0.07	362.50	0.05	360.00	0.10	352.17	0.73	352.17

>95 years	0.01	366.67	0.01	600.00	0.03	400.00	0.14	403.70
Early-onset	1.17	144.89	1.56	155.74	0.97	137.16	45.75	137.27
Later-onset	7.94	147.91	8.37	153.34	7.97	146.77	183.02	136.62
Total	9.11	147.50	9.93	153.87	8.95	145.81	228.77	136.75

Supplementary Table S9. The percentage change of GBTC cases from 1990 to 2019 by SDI and age in females

Socio-demographic index (SDI)	Incidence		Prevalence		Deaths		DALYs	
	Number in 2019 ×10[3]	Percentage change in Absolute number (%)	Number in 2019 ×10[3]	Percentage change in Absolute number (%)	Number in 2019 ×10[3]	Percentage change in Absolute number (%)	Number in 2019 ×10[3]	Percentage change in Absolute number (%)
**High SDI**								
20-24 years	0.01	-23.53	0.04	-10.26	0.004	-42.86	0.28	-40.94
25-29 years	0.02	-32.26	0.06	-20.29	0.01	-42.86	0.48	-45.48
30-34 years	0.05	-25.35	0.13	-14.67	0.02	-41.67	1.23	-40.23
35-39 years	0.12	-24.38	0.29	-12.23	0.05	-39.29	2.68	-38.73
40-44 years	0.24	-25.54	0.55	-14.80	0.11	-38.33	5.27	-37.96
45-49 years	0.40	-23.22	0.80	-13.30	0.25	-32.14	10.44	-32.03
50-54 years	0.72	-22.63	1.27	-14.47	0.50	-29.49	18.58	-29.38
55-59 years	1.33	-17.06	2.28	-5.72	0.88	-26.67	28.79	-26.53
60-64 years	2.07	-16.11	3.36	-5.51	1.45	-24.69	40.80	-24.49
65-69 years	3.03	-18.26	4.85	-7.69	2.09	-26.54	49.55	-26.36
70-74 years	4.08	3.68	6.35	16.63	2.97	-5.38	57.65	-5.12
75-79 years	5.11	0.65	7.43	16.73	3.87	-9.51	59.34	-9.45
80-84 years	5.86	29.47	8.34	50.16	4.53	18.49	53.39	18.28
85-89 years	5.91	105.63	7.54	138.14	4.62	92.27	41.76	91.37
90-94 years	3.49	284.34	3.37	343.36	3.26	259.82	22.80	257.32
>95 years	1.47	659.28	0.77	671.00	1.85	624.71	9.52	599.12
Early-onset	0.85	-24.44	1.85	-13.85	0.44	-35.47	20.38	-35.59
Later-onset	33.07	26.12	45.56	33.61	26.01	21.15	382.18	-1.45
Total	33.92	24.04	47.41	30.80	26.45	19.41	402.56	-4.03
**High-middle SDI**								
20-24 years	0.02	-25.81	0.05	-15.52	0.01	-38.89	0.73	-38.21
25-29 years	0.04	-18.87	0.09	-5.38	0.02	-30.30	1.41	-29.93

30-34 years	0.10	-1.01	0.18	14.29	0.06	-16.18	3.25	-15.71
35-39 years	0.20	-4.76	0.36	11.73	0.12	-19.33	6.32	-19.13
40-44 years	0.39	10.76	0.68	28.84	0.25	-4.92	11.82	-4.81
45-49 years	0.71	28.52	1.13	40.62	0.55	19.26	23.02	19.44
50-54 years	1.33	26.64	1.97	35.35	1.08	20.29	40.37	20.25
55-59 years	2.16	27.06	3.04	35.41	1.73	20.53	56.52	20.62
60-64 years	3.16	20.11	4.19	26.23	2.65	15.55	74.58	15.62
65-69 years	4.05	30.93	5.23	40.06	3.41	24.44	80.41	24.42
70-74 years	4.25	48.88	5.36	62.68	3.72	40.03	71.82	40.17
75-79 years	3.96	20.94	4.66	34.73	3.61	13.26	55.25	13.09
80-84 years	3.86	69.06	4.42	92.92	3.58	56.36	42.14	55.93
85-89 years	2.88	144.35	2.99	189.36	2.68	120.53	24.16	119.98
90-94 years	1.19	287.25	1.00	355.71	1.25	244.88	8.74	243.40
>95 years	0.28	405.36	0.15	403.45	0.39	358.14	2.09	348.50
Early-onset	1.47	12.85	2.49	26.73	1.01	1.82	46.55	-0.02
Later-onset	27.11	47.15	33.00	56.57	24.08	40.40	456.07	30.06
Total	28.58	44.89	35.49	54.03	25.09	38.31	502.62	26.53
**Middle SDI**								
20-24 years	0.04	-18.37	0.08	-8.24	0.02	-26.67	1.45	-28.14
25-29 years	0.08	-1.28	0.14	10.94	0.05	-13.46	2.80	-13.09
30-34 years	0.15	25.00	0.25	42.44	0.09	9.30	5.38	10.18
35-39 years	0.29	25.76	0.47	45.37	0.19	9.04	10.04	9.15
40-44 years	0.52	56.33	0.81	81.94	0.37	35.66	17.40	36.12
45-49 years	0.95	83.59	1.40	98.31	0.77	72.32	32.54	72.12
50-54 years	1.79	101.58	2.50	113.68	1.51	92.87	56.65	92.92
55-59 years	2.49	96.61	3.27	109.74	2.10	86.73	68.62	86.80
60-64 years	3.37	106.63	4.19	120.59	2.97	96.88	83.31	96.82
65-69 years	3.91	127.15	4.64	147.05	3.50	113.68	82.57	113.58
70-74 years	3.79	132.19	4.28	155.46	3.58	117.70	69.17	117.79
75-79 years	3.29	143.05	3.38	171.38	3.29	126.20	50.30	125.76
80-84 years	2.66	210.04	2.57	249.59	2.77	186.44	32.62	185.94

85-89 years	1.55	253.08	1.32	297.29	1.68	223.75	15.15	223.58
90-94 years	0.49	390.00	0.35	447.62	0.61	351.11	4.27	350.11
>95 years	0.12	505.26	0.06	490.00	0.19	466.67	0.98	453.11
Early-onset	2.02	52.80	3.15	69.14	1.50	40.38	69.60	36.47
Later-onset	23.44	136.71	26.56	151.26	22.18	126.32	463.63	112.34
Total	25.45	126.78	29.70	138.94	23.68	117.94	533.24	97.97
**Low-middle**								
20-24 years	0.06	78.13	0.10	87.04	0.04	75.00	2.35	73.32
25-29 years	0.10	88.24	0.16	96.25	0.06	77.78	3.95	79.13
30-34 years	0.16	111.69	0.24	122.02	0.12	103.39	6.86	102.75
35-39 years	0.30	118.98	0.43	130.98	0.23	109.09	11.98	108.91
40-44 years	0.52	144.34	0.69	159.18	0.43	134.81	20.00	134.96
45-49 years	0.93	141.82	1.27	148.63	0.81	137.35	33.98	137.06
50-54 years	1.69	170.03	2.22	175.97	1.50	166.67	55.99	166.71
55-59 years	2.15	152.00	2.64	158.68	1.91	148.18	62.41	148.42
60-64 years	2.77	153.30	3.21	160.60	2.57	150.10	72.12	150.07
65-69 years	2.93	181.08	3.18	191.21	2.79	176.53	65.97	176.42
70-74 years	2.73	182.97	2.79	194.61	2.75	178.06	53.22	178.11
75-79 years	2.09	193.82	1.93	208.32	2.25	187.61	34.49	186.73
80-84 years	1.52	271.64	1.31	290.72	1.72	263.42	20.33	262.64
85-89 years	0.73	320.69	0.55	336.51	0.87	309.95	7.84	310.15
90-94 years	0.21	444.74	0.13	454.17	0.28	426.42	1.95	424.46
>95 years	0.05	466.67	0.03	550.00	0.09	480.00	0.45	460.49
Early-onset	2.07	130.98	2.89	139.62	1.68	125.34	79.11	122.72
Later-onset	16.85	184.93	17.97	189.62	16.71	183.80	374.76	171.66
Total	18.92	177.83	20.85	181.40	18.40	177.30	453.87	161.64
**Low SDI**								
20-24 years	0.02	133.33	0.04	164.29	0.01	116.67	0.90	133.68
25-29 years	0.04	125.00	0.06	137.50	0.03	108.33	1.53	114.27
30-34 years	0.06	142.31	0.09	160.00	0.05	128.57	2.76	133.47
35-39 years	0.12	141.67	0.16	154.84	0.09	132.50	4.83	134.06

40-44 years	0.19	162.16	0.25	178.65	0.17	153.85	7.78	154.20
45-49 years	0.34	141.55	0.46	147.83	0.30	138.58	12.77	137.64
50-54 years	0.55	141.85	0.71	147.39	0.49	139.32	18.46	139.22
55-59 years	0.70	132.01	0.85	138.31	0.63	129.35	20.72	129.02
60-64 years	0.89	147.90	1.01	155.73	0.83	145.00	23.42	145.12
65-69 years	0.92	166.18	0.97	176.92	0.90	163.24	21.14	162.61
70-74 years	0.85	172.35	0.84	184.35	0.87	169.14	16.87	168.77
75-79 years	0.62	186.05	0.54	201.12	0.68	183.75	10.44	182.24
80-84 years	0.39	247.75	0.31	263.95	0.45	244.27	5.34	242.33
85-89 years	0.17	295.35	0.12	313.79	0.21	292.45	1.88	287.65
90-94 years	0.05	327.27	0.03	383.33	0.07	340.00	0.46	337.14
>95 years	0.01	450.00	0.01	500.00	0.02	375.00	0.10	415.00
Early-onset	0.77	145.40	1.05	156.62	0.65	138.75	30.57	139.21
Later-onset	5.13	166.56	5.38	171.38	5.15	167.03	118.82	155.48
Total	5.91	163.63	6.42	168.67	5.80	163.55	149.39	151.97

Supplementary Table S10. The percentage change of GBTC cases from 1990 to 2019 by SDI and age in males

Socio-demographic index (SDI)	Incidence		Prevalence		Deaths		DALYs	
	Number in 2019 ×10³	Percentage change in Absolute number (%)	Number in 2019 ×10³	Percentage change in Absolute number (%)	Number in 2019 ×10³	Percentage change in Absolute number (%)	Number in 2019 ×10³	Percentage change in Absolute number (%)
**High SDI**								
20-24 years	0.02	-11.76	0.04	-2.38	0.004	-33.33	0.28	-32.10
25-29 years	0.03	-22.22	0.07	-14.81	0.01	-35.71	0.56	-37.17
30-34 years	0.08	-9.20	0.19	-1.05	0.03	-23.68	1.69	-22.31
35-39 years	0.16	-12.37	0.38	-4.27	0.06	-25.00	3.29	-25.31
40-44 years	0.29	-20.72	0.68	-11.33	0.13	-32.47	6.21	-32.19
45-49 years	0.48	-9.16	0.85	2.54	0.27	-21.16	11.53	-20.91
50-54 years	0.83	-7.68	1.49	6.60	0.54	-19.52	20.11	-19.39
55-59 years	1.57	3.70	3.09	21.49	0.97	-10.82	31.98	-10.62
60-64 years	2.37	15.49	4.24	33.63	1.57	1.36	44.32	1.61
65-69 years	3.56	32.14	6.25	51.25	2.34	17.81	55.48	18.02
70-74 years	4.67	75.44	7.40	99.81	3.28	59.21	63.63	59.31
75-79 years	5.22	74.54	8.13	100.42	3.85	60.79	59.27	60.55
80-84 years	5.04	136.11	7.75	172.00	3.89	119.14	46.17	118.19
85-89 years	3.85	243.48	5.13	282.20	3.07	235.52	27.92	233.34
90-94 years	1.45	417.92	1.35	435.18	1.47	427.34	10.31	423.03
>95 years	0.32	695.00	0.17	685.71	0.48	743.86	2.50	718.30
Early-onset	1.05	-13.53	2.21	-4.25	0.51	-25.40	23.55	-25.49
Later-onset	28.87	76.21	45.00	91.73	21.45	68.03	361.69	39.10
Total	29.91	70.02	47.21	83.14	21.96	63.31	385.24	32.10
**High-middle SDI**								
20-24 years	0.02	-25.00	0.04	-14.29	0.01	-37.50	0.67	-37.20
25-29 years	0.05	8.51	0.10	23.08	0.03	-6.90	1.69	-6.32

30-34 years	0.16	47.22	0.27	68.10	0.09	25.68	5.32	26.59
35-39 years	0.27	30.43	0.46	50.66	0.16	10.14	8.51	10.84
40-44 years	0.49	65.77	0.89	87.95	0.32	44.55	15.00	44.91
45-49 years	0.72	87.08	0.99	101.02	0.55	75.40	23.17	75.33
50-54 years	1.30	83.83	1.82	94.96	1.05	75.97	39.18	76.04
55-59 years	2.00	79.51	3.03	93.62	1.58	70.01	51.65	70.00
60-64 years	2.65	77.56	3.69	91.00	2.19	69.40	61.73	69.49
65-69 years	3.32	109.54	4.50	128.93	2.74	97.33	64.79	97.06
70-74 years	3.40	141.01	4.21	166.08	2.95	126.00	57.09	125.63
75-79 years	3.01	112.52	3.66	139.80	2.72	98.54	41.85	98.05
80-84 years	2.57	192.83	3.18	243.04	2.38	167.30	28.15	165.78
85-89 years	1.74	325.18	1.94	410.82	1.63	281.07	14.83	279.74
90-94 years	0.43	403.53	0.38	476.92	0.46	357.43	3.27	356.92
>95 years	0.07	490.91	0.03	466.67	0.09	447.06	0.50	427.66
Early-onset	1.72	59.91	2.75	76.30	1.16	45.00	54.36	41.88
Later-onset	20.48	124.91	26.44	142.93	17.79	113.92	363.04	97.64
Total	22.20	118.07	29.18	134.60	18.95	107.88	417.39	88.02
**Middle SDI**								
20-24 years	0.04	-2.70	0.07	11.29	0.02	-17.39	1.25	-17.97
25-29 years	0.07	21.43	0.12	42.35	0.04	2.70	2.39	4.64
30-34 years	0.18	58.93	0.29	85.81	0.11	36.59	6.36	36.14
35-39 years	0.33	56.94	0.52	87.00	0.21	31.68	11.06	32.32
40-44 years	0.62	115.79	1.03	160.86	0.42	81.03	19.80	81.29
45-49 years	0.93	147.20	1.20	170.81	0.73	127.95	31.00	127.91
50-54 years	1.58	161.32	2.12	179.79	1.32	146.92	49.44	147.13
55-59 years	2.23	159.12	3.16	181.96	1.85	143.04	60.65	142.97
60-64 years	2.74	164.32	3.53	186.29	2.40	148.96	67.53	149.16
65-69 years	3.06	192.17	3.72	221.05	2.74	172.27	64.74	172.29
70-74 years	2.98	205.43	3.26	236.67	2.83	184.78	54.67	184.50
75-79 years	2.54	229.96	2.64	269.19	2.57	206.56	39.43	205.74
80-84 years	1.79	302.02	1.79	350.25	1.90	271.09	22.48	269.96

85-89 years	0.99	354.84	0.86	407.65	1.09	318.77	9.92	318.69
90-94 years	0.24	412.77	0.17	461.29	0.31	379.69	2.17	378.37
>95 years	0.05	557.14	0.02	500.00	0.08	500.00	0.40	485.51
Early-onset	2.15	100.37	3.23	127.66	1.54	79.11	71.86	73.73
Later-onset	18.20	202.69	21.28	224.61	17.08	187.27	371.44	171.26
Total	20.36	187.18	24.50	207.38	18.62	173.66	443.30	148.64
**Low-middle**								
20-24 years	0.02	64.29	0.04	86.36	0.01	44.44	0.90	55.46
25-29 years	0.04	86.36	0.07	100.00	0.03	66.67	1.56	70.26
30-34 years	0.10	120.00	0.15	139.34	0.07	109.09	3.92	105.88
35-39 years	0.19	130.86	0.26	149.52	0.14	112.50	7.06	112.20
40-44 years	0.33	142.65	0.48	165.75	0.26	124.56	12.05	123.72
45-49 years	0.54	145.05	0.65	156.08	0.46	134.36	19.30	134.76
50-54 years	0.89	142.08	1.12	150.78	0.77	134.95	28.92	134.71
55-59 years	1.24	144.09	1.63	154.60	1.08	135.73	35.44	135.76
60-64 years	1.51	124.22	1.81	134.54	1.38	117.11	38.91	117.06
65-69 years	1.67	158.54	1.87	173.75	1.58	149.92	37.30	149.57
70-74 years	1.54	168.35	1.55	183.03	1.55	159.46	30.02	159.07
75-79 years	1.26	187.70	1.19	205.38	1.36	177.35	20.87	176.64
80-84 years	0.81	240.59	0.73	261.58	0.93	228.72	10.98	227.75
85-89 years	0.41	321.43	0.32	337.84	0.50	309.09	4.50	307.60
90-94 years	0.11	465.00	0.07	469.23	0.16	437.93	1.10	442.08
>95 years	0.02	500.00	0.01	800.00	0.03	540.00	0.17	507.14
Early-onset	1.22	135.38	1.65	150.99	0.96	122.33	44.79	120.27
Later-onset	9.46	165.47	10.32	173.49	9.33	160.70	208.22	147.74
Total	10.69	161.66	11.97	170.15	10.29	156.52	253.01	142.39
**Low SDI**								
20-24 years	0.01	175.00	0.02	200.00	0.01	250.00	0.46	194.19
25-29 years	0.01	180.00	0.02	175.00	0.01	125.00	0.58	156.39
30-34 years	0.03	166.67	0.04	175.00	0.02	155.56	1.30	153.50
35-39 years	0.06	148.00	0.08	164.52	0.05	135.00	2.44	140.83

40-44 years	0.11	144.19	0.14	166.67	0.09	132.43	4.04	133.47
45-49 years	0.17	128.95	0.20	138.82	0.15	122.06	6.36	122.00
50-54 years	0.27	111.63	0.34	118.06	0.24	105.04	9.12	105.17
55-59 years	0.37	101.09	0.47	109.29	0.33	94.12	10.80	94.56
60-64 years	0.47	96.62	0.55	104.48	0.44	92.11	12.33	91.92
65-69 years	0.51	111.52	0.56	123.90	0.50	106.20	11.81	105.62
70-74 years	0.48	116.74	0.47	127.80	0.49	111.11	9.58	110.79
75-79 years	0.37	137.01	0.33	148.87	0.40	130.29	6.20	130.13
80-84 years	0.21	180.00	0.18	193.55	0.25	175.28	2.91	173.40
85-89 years	0.10	277.78	0.08	285.00	0.13	281.82	1.14	274.43
90-94 years	0.03	350.00	0.02	466.67	0.04	375.00	0.27	378.57
>95 years	0.00	200.00	0.002	NA	0.01	500.00	0.03	371.43
Early-onset	0.40	141.21	0.51	156.50	0.32	130.71	15.18	133.39
Later-onset	2.81	119.98	2.997	126.53	2.82	117.32	64.20	108.18
Total	3.21	122.41	3.51	130.62	3.15	118.69	79.38	112.58

Supplementary Figure S1

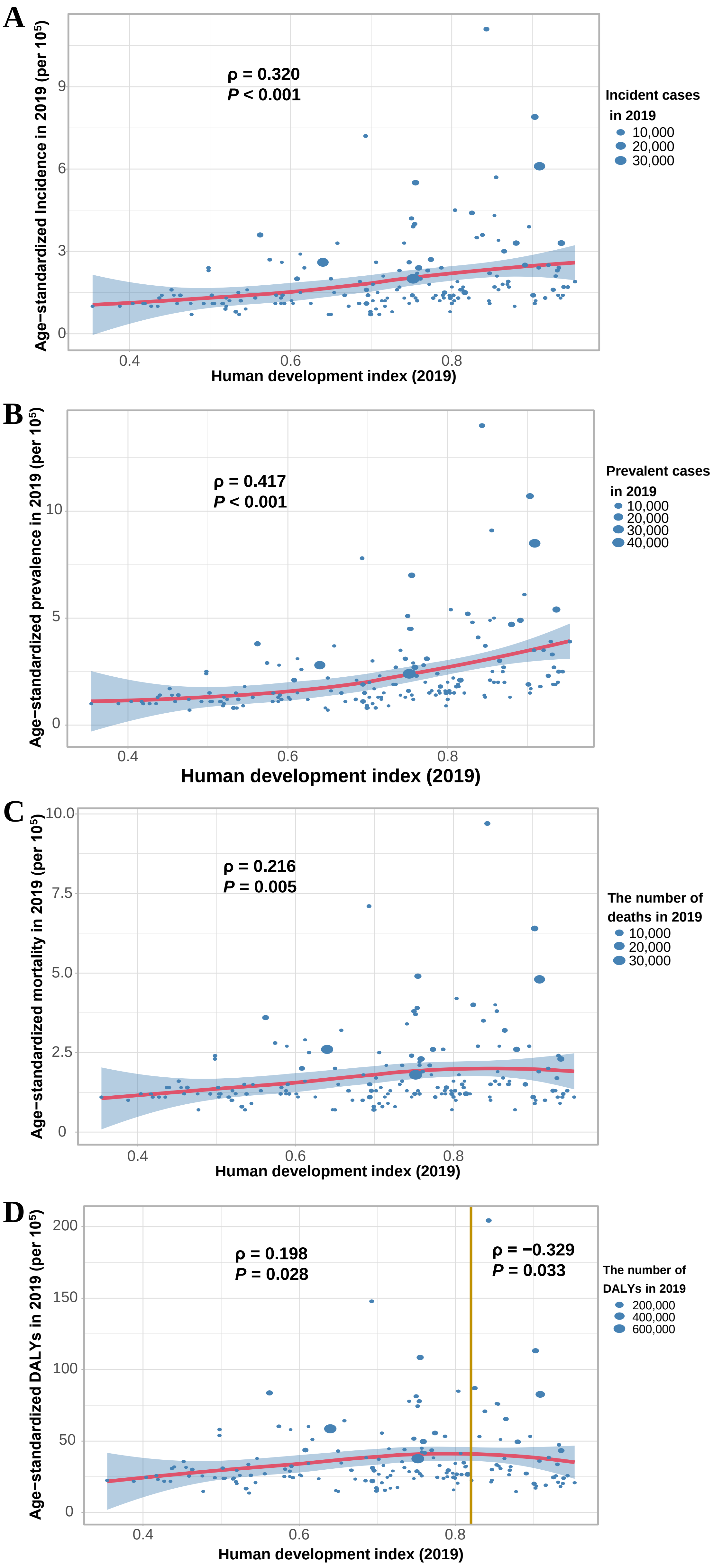

Supplementary Figure S2

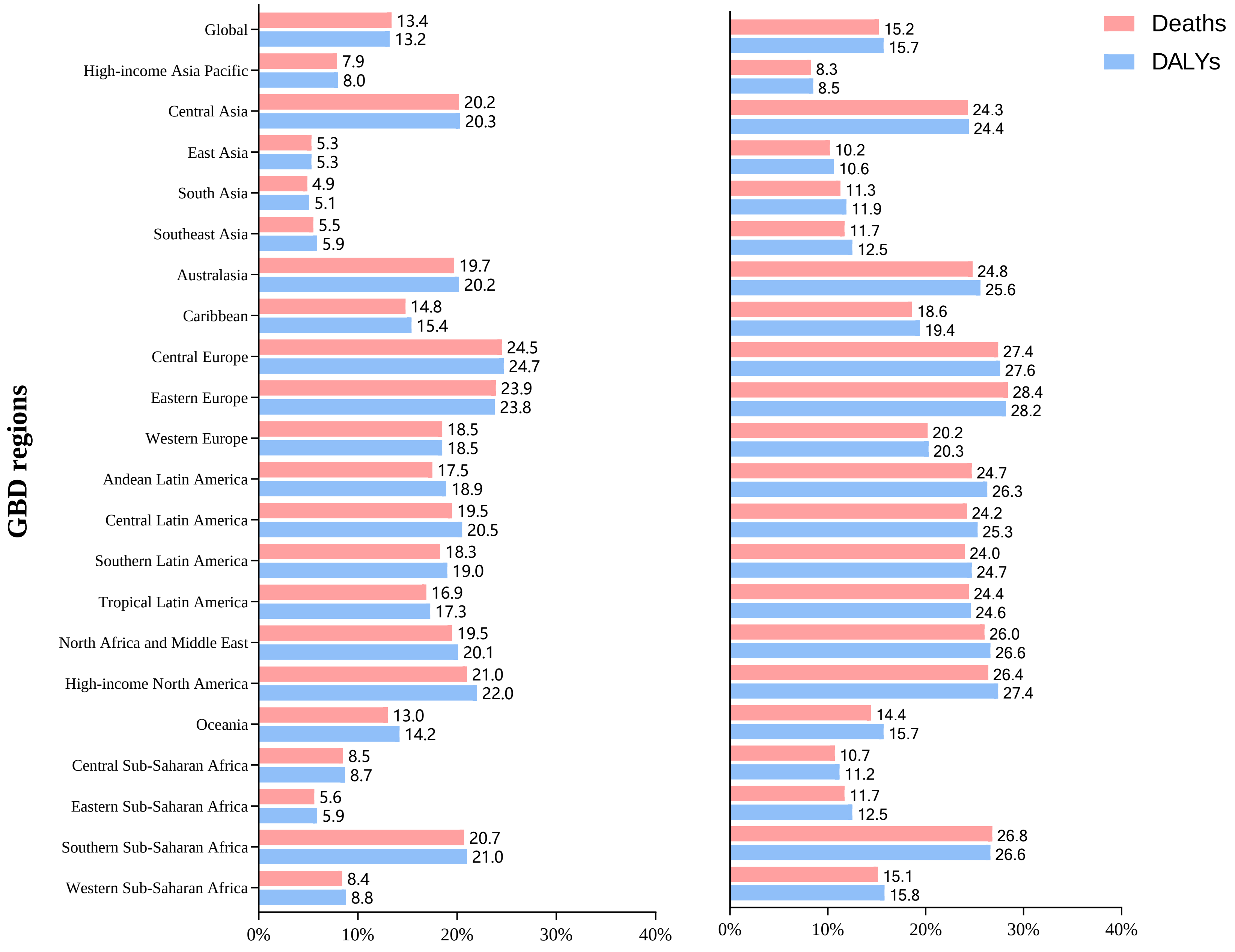

Supplement: Supplementary file 1 [file Data_Sheet_1.PDF]
